# Network Pharmacology-Based Analysis of Xiao-Xu-Ming Decoction on the Treatment of Alzheimer’s Disease

**DOI:** 10.3389/fphar.2020.595254

**Published:** 2020-12-04

**Authors:** Yanjia Shen, Baoyue Zhang, Xiaocong Pang, Ran Yang, Miao Chen, Jiaying Zhao, Jinhua Wang, Zhe Wang, Ziru Yu, Yuehua Wang, Li Li, Ailin Liu, Guanhua Du

**Affiliations:** Beijing Key Laboratory of Drug Targets Identification and Drug Screening, Institute of Materia Medica, Chinese Academy of Medical Sciences and Peking Union Medical College, Beijing, China

**Keywords:** Alzheimer's disease, Xiao-Xu-Ming decoction, network pharmacology, machine learning, molecular docking

## Abstract

Alzheimer's disease (AD) has become a worldwide disease that is harmful to human health and brings a heavy economic burden to healthcare system. Xiao-Xu-Ming Decoction (XXMD) has been widely used to treat stroke and other neurological diseases for more than 1000 years in China. However, the synergistic mechanism of the constituents in XXMD for the potential treatment of AD is still unclear. Therefore, the present study aimed to predict the potential targets and uncover the material basis of XXMD for the potential treatment of AD. A network pharmacology-based method, which combined data collection, drug-likeness filtering and absorption, distribution, metabolism, excretion and toxicity (ADME/T) properties filtering, target prediction and network analysis, was used to decipher the effect and potential targets of XXMD for the treatment of AD. Then, the acetylcholinesterase (AChE) inhibitory assay was used to screen the potential active constituents in XXMD for the treatment of AD, and the molecular docking was furtherly used to identify the binding ability of active constituents with AD-related target of AChE. Finally, three *in vitro* cell models were applied to evaluate the neuroprotective effects of potential lead compounds in XXMD. Through the China Natural Products Database, Traditional Chinese Medicine Systems Pharmacology (TCMSP) Database, Traditional Chinese Medicine (TCM)-Database @Taiwan and literature, a total of 1481 compounds in XXMD were finally collected. After ADME/T properties filtering, 908 compounds were used for the further study. Based on the prediction data, the constituents in XXMD formula could interact with 41 AD-related targets. Among them, cyclooxygenase-2 (COX-2), estrogen receptor α (ERα) and AChE were the major targets. The constituents in XXMD were found to have the potential to treat AD through multiple AD-related targets. 62 constituents in it were found to interact with more than or equal to 10 AD-related targets. The prediction results were further validated by *in vitro* biology experiment, resulting in several potential anti-AD multitarget-directed ligands (MTDLs), including two AChE inhibitors with the IC_50_ values ranging from 4.83 to 10.22 μM. Moreover, fanchinoline was furtherly found to prevent SH-SY5Y cells from the cytotoxicities induced by sodium nitroprusside, sodium dithionate and potassium chloride. In conclusion, XXMD was found to have the potential to treat AD by targeting multiple AD-related targets and canonical pathways. Fangchinoline and dauricine might be the potential lead compounds in XXMD for the treatment of AD.

## Introduction

Alzheimer's disease (AD) is a most common degenerative disease of the central nervous system, characterized by memory loss, cognitive dysfunction, loss of acquired learning ability, nausea, etc. It has become a worldwide disease that is harmful to human health. According to the data from the International Alzheimer's Association in 2018, there were 50 million people worldwide suffering from AD in 2018. It is estimated to increase to 152 million AD patients worldwide by 2050 (Patterson, 2018). Over the last two decades, the neuropathological features of ad are recognized. However, the pathological mechanisms of AD have not been clearly defined. The non-availability of effective treatment which can prevent the onset and progression of AD may be caused by the lack of understanding of the pathogenic process. Up to now, four cholinesterase inhibitors including tacrine, donepezil, rivastigmine and galantamine have been approved for the treatment of AD clinically. These drugs were found to slow down the disease progression, providing symptomatic relief but failed to achieve a definite cure. More and more evidences suggest that AD is a multifactorial disease, therefore the traditional therapies that targeting one single target is not suitable. Hence, multi-targets drugs might be another choice for the treatment of AD.

Traditional Chinese Medicine (TCM), with a history of more than three thousand years, is based on the concept of “multiple components and multiple targets” and there are many herbal prescriptions used for the treatment of AD (Liu et al., 2014). It is a good way to screen TCM to obtain the novel potential neuroprotective agents by evaluating the neuroprotective effects on the *in vivo* and *in vitro* experimental models of AD. Xiao-Xu-Ming Decoction (XXMD), which had been firstly published in the “Preparation for Emergency Medicine” written by Sun Simiao in the Tang Dynasty, has long been used clinically to treat stroke and has notable effects. XXMD consists of twelve herbs, including Huang Qin (*Scutellaria baicalensis Georgi*), Shao Yao (*Paeonia lactiflora Pall.*), Gan Cao (*Glycyrrhiza uralensis Fisch. ex DC.*), Fang Ji (*Stephania tetrandra S.Moore*), Ren Shen (*Panax ginseng C.A.Mey.*), Gui Zhi (*Cinnamomum cassia (L.) J.Presl*), Xin Ren (*Prunus armeniaca L.*), Ma Huang (*Ephedra sinica Stapf*), Chuan Xiong (*Conioselinum anthriscoides “Chuanxiong”*), Fu Zi (*Aconitum carmichaeli Debeaux*), Fang Feng (*Saposhnikovia divaricata (Turcz. ex Ledeb.) Schischk.*) and Sheng Jiang (*Zingiber officinale Roscoe*) at a ratio of 1: 1: 1: 1: 1: 1: 1: 1: 1: 1: 1.5: 5. The preliminary studies of XXMD are mainly focused on its neuroprotective effects to stroke. Several studies have suggested that XXMD alleviates blood−brain barrier (BBB) dysfunction and protects mitochondria and neurovascular unit from cerebral injury induced by cerebral ischemia and reperfusion (Lan et al., 2013a; Lan et al., 2013b; Lan et al., 2014). Learning and memory improvement ability of XXMD has also been reported in rats with chronic cerebral ischemia or aging (Wang and Du, 2006; Wang et al. 2012). Moreover, through high throughput screening methods, several components of XXMD have been reported to have the potential effects on anti-Aβ neurotoxicity, anti-H_2_O_2_ damage, anti-glutamic acid damage and β-secretase activity. So, the combination of these components was regarded as the active component combination of XXMD for anti-AD effects (Wang and Du, 2005). Based on high performance liquid chromatography (HPLC), six constituents with a demonstrated pharmacological activity in the anti-AD active component group of XXMD were determined, and the six components were paeoniflorin, baicalin, fangchinoline, tetrandrine, prim-O-glucosylcimifugin and 4'-O-beta-D-glucosyl-5-O-methylvisamminol (Li et al., 2007). The neuroprotective effects of the six active constituents on the treatment of AD have been widely studied (He et al., 2011; Chen et al., 2015; Gu X. et al. 2016). Therefore, XXMD has potential therapeutic effects for treating AD. However, its AD-treating material basis and targets still are unclear.

With the rapid progress in bioinformatics, systems biology and poly-pharmacology, network-based drug discovery are considered as promising approaches for cost-effective drug development. Network pharmacology centers on the complicated interactions in biological systems from a holistic perspective, rather than altering the single molecular component. It has great advantages in identifying alternative targets for herbal medicines, discovering multi-target drugs and providing a new insight for studying TCM (Zhang et al., 2019).

In this study, all the herb constituents in XXMD were obtained from three online databases shown in methods and literature. Then the drug-likeness analysis was carried out to filter the constituents for further study. Subsequently, two kinds of fingerprints (ECFP_6 and MACCS) and two machine learning algorithms (naive Bayesian and recursive partitioning), which have been established previously (Fang et al., 2015), were applied to predict the potential active compounds identification and targets of XXMD for treating AD. Finally, several available constituents from XXMD were used in *in vitro* experimental validation to assess their actual effects on the treatment of AD.

## Materials and Methods

### Data Collection and Preparation

The chemical structures in XXMD were collected from the China Natural Product Database (http://pharmdata.ncmi.cn), TCMSP (Traditional Chinese Medicine Systems Pharmacology, http://lsp.nwsuaf.edu.cn/tcmsp.php) Database, TCM-Database @Taiwan (http://tcm.cmu.edu.tw) and literatures. Then, the data were further filtered basing upon Lipinski’s rules (Lipinski et al., 2001). To discover the potential chemical compositions against AD, the data were filtered basing on the properties of absorption, distribution, metabolism, excretion and toxicity (ADME/T) before target prediction. The following properties, including human intestinal absorption (HIA), aqueous solubility, BBB penetration and cytochrome P450 (CYP450) 2D6 inhibition were predicted by using the ADME/T descriptors module available in Discovery Studio 2016. ADME/T descriptors calculated the following related properties and the following constituents were rejected basing on the predicted results: 1) Solubility < -8.0, extremely low; 2) BBB = 3, low penetrant; 3) CYP2D6, TRUE; 4) Absorption = 3, very low absorption.

### Target Prediction

Two machine learning tools (naive Bayesian and recursive partitioning) and two kinds of fingerprint descriptors (ECFP6 and MACCS) which had been developed previously were applied to predict the potential targets of XXMD for the prevention of AD (Fang et al., 2015). At first, two machine learning algorithms (naive Bayesian and recursive segmentation) were used to construct a drug target prediction platform (AlzhCPI, http://rcidm.org/AlzhCPI/index.html) (Fang et al., 2017a) for AD. AlzhCPI can predict the activity of 52 key targets related to AD. Then, the AlzhCPI was successfully applied in the discovery of multi-target anti-AD lead compounds. A compound is defined as positive if the compound was predicted to be active by at least three out of four single classifiers.

### Network Construction and Analysis

To explore the potential effective constituents in the XXMD formula, the drug distribution and biological function process of candidate constituents were carried out. To study the possible biological pathways of the constituents and relations between the targets, GO (http://www.geneontology.org/) and KEGG (http//www.genome.jp/kegg) analysis through the Database of Annotation, Visualization and Integrated Discovery (DAVID) database were performed. After that, the Search Tool for the Retrieval of Interacting Genes/Proteins (STRING) was used to establish interactions between targets. To evaluate the therapeutic mechanisms of the XXMD formula in treating AD, constituent-target network (C-T network), target-target network (T-T network) and target-function network (T-F network) were constructed. In these networks, the nodes represent constituents, targets or functional modules and edges represent links between them. To visualize C-T, T-T and T-F networks, Cytoscape 3.7.1 was used in the study.

### *In vitro* acetylcholinesterase Inhibitory Assay

The acetylcholinesterase (AChE) inhibitory activity was assessed by the Ellman’s method (Ellman et al., 1961). Donepezil, an AChE inhibitor widely used for the treatment of AD, was used as the reference compound. The substrate ASCh and 5, 5′-Dithiobis (2-nitrobenzoic acid) (DTNB) were bought from Sigma Aldrich and AChE was obtained from brains of the SD rats. The blood vessels were removed from brains and then the brains were homogenized with 20-fold 0.9% saline solution on the ice. After centrifuged for 20 min at 800 × g at 4°C, the supernatant from brain homogenate was collected. To determine the inhibition of AChE activity, six serial dilutions of samples were added. The reaction system includes 10 µl sample, 30 µl 0.05 mol/L phosphate-buffered solution (PBS), 20 µl AChE solution, 60 µl 3.75 mmol/L ASCh, and 80 µl 0.25 mg/ml DTNB and was incubated for 60 min at 37°C. Then the absorbance intensity of AChE reaction system was quantified at 412 nm by Spectra Max M5 microplate reader (Molecular Devices, Sunnyvale, CA) (Zhou et al., 2016). Experiments were in accordance with guidelines for animal care and were approved by Animal Ethics Committee of Chinese Academy of Medical Sciences & Peking Union Medical College.

### Target Identification Based on Molecular Docking

To identify the binding ability of active constituents with AD-related targets, the crystal structures of AChE (PDB code: 4EY7) were obtained from RCSB Protein Data Bank (http://www.pdb.org/) to establish molecular docking model with Discovery Studio 2016.

By using the tool of Prepare Ligands, a ligand library which contains conformations of each ligand was built. Then, the ligands and water of crystallization in target proteins were removed through the tool of Prepare Protein. Through the tool of Define and Edit Binding Site, the binding sites which contact receptors and ligands were searched according to the information of PDB or the sites of the original ligands of the proteins.

The CDOCKER module of Dock Ligands in Discovery Studio 2016 was used to do the docking. The Conformation Method was set as Fast to make the docking accuracy and velocity, and the other parameters were set as default.

### Cell-Based Assays

#### Cell Culture and Treatment

Human neuroblastoma SH-SY5Y cells were maintained in a medium consisting of DMEM supplemented with 10% fetal bovine serum (FBS, Gibco, Australia) in humidified 5% CO2 at 37°C. SH-SY5Y cells were plated at a density of 5,000 cells per well in 96-well plates and cultured for 19 h. To determine the neuroprotective effect of fangchinoline on SH-SY5Y cells, cells were pre-treated with 0.125, 0.25, 0.5, and 1 μM fangchinoline for 2 h and then treated with sodium nitroprusside (500 μM), sodium dithionate (8 mM) or potassium chloride (80 mM) for 24 h, respectively.

#### MTT Assay

Cell viability was examined by MTT (3-[4,5-dimethyl-2-thiazolyl]-2,5-diphenyl-2-tetrazolium bromide). Briefly, after treatment of sodium nitroprusside (Sigma), sodium dithionate (Sigma, USA) or potassium chloride, 100 µl of MTT (0.5 mg/ml in medium) was added to each well and then incubated at 37°C for 4 h. The supernatant was removed carefully and 100 µl of dimethyl sulfoxide (DMSO) was added to each well and the absorbance at 490 nm was measured with Spectra Max M5 microplate reader (Molecular Devices, Sunnyvale, CA).

### Statistical Methods

All data are presented as mean ± SEM. Statistical analysis was carried out using the Graph Pad Prism version 7.0 software and the significance of each group was verified with one-way analysis of variance (ANOVA) followed by Tukey's multiple comparison post hoc test. A *P* value <0.05 was considered significant.

## Results

### Property Analysis of Constituents in Xiao-Xu-Ming Decoction Formula

**FIGURE 1:**
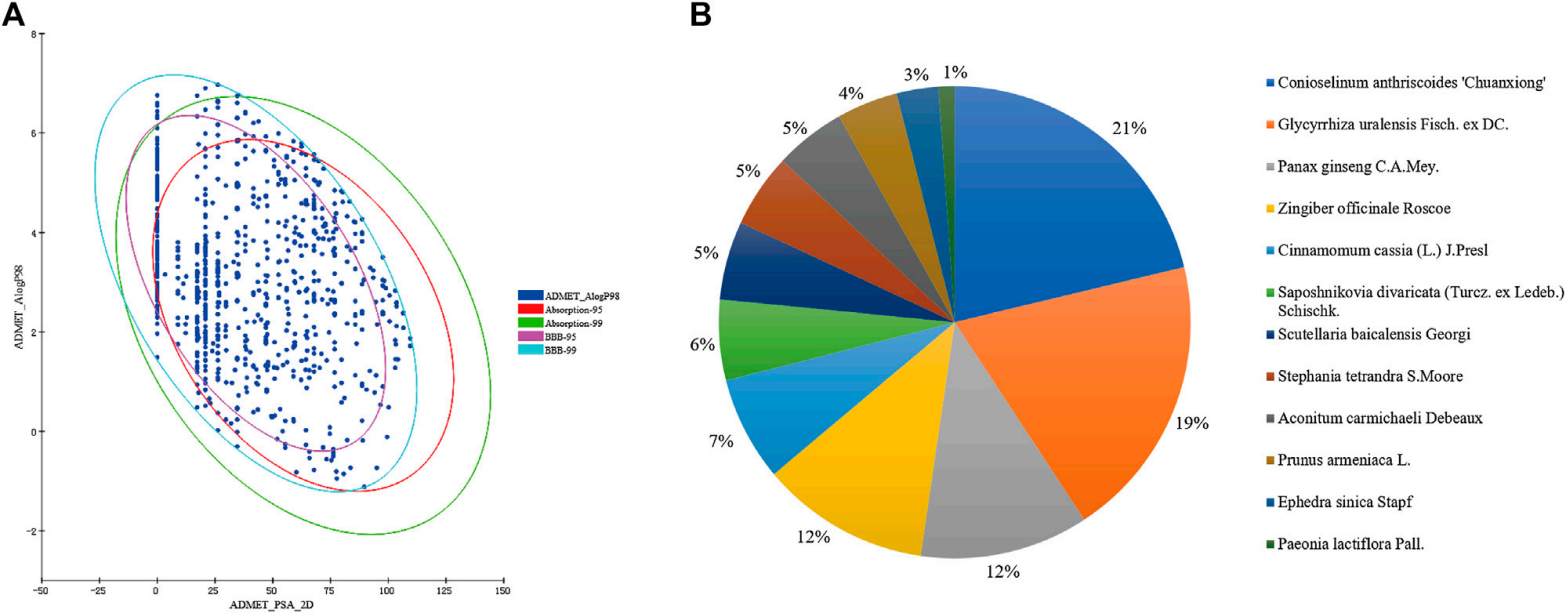
Prediction of blood−brain barrier (BBB) penetration and human intestinal absorption of the constituents in XXMD **(A)** and herb distribution of candidate constituents in XXMD **(B)**.

Through the China Natural Products database, TCMSP database, TCM-Database @Taiwan and literature, a total of 1481 compounds from twelve herbs in XXMD formula were finally collected. ADME/T characteristics play a significant role in the drug-likeness property of a compound. As most traditional Chinese medicines are administered orally in combination, also BBB is an important factor which affects the function of drugs on central nervous system, to filter out constituents with undesirable properties and increase the possibilities of turning drug candidates into drugs at the later stages of drug discovery, drug-likeness filtering and ADME/T filtering were applied. After the drug-likeness filtering and ADME/T filtering, 908 compounds were kept for further study. The ADME/T filtering results of constituents in XXMD were shown in Figure 1A. The results suggest that only about one-third of constituents of XXMD have good BBB permeability. Therefore, these part of constituents that could penetrate the BBB might be the main constituents of XXMD that exert the anti-AD effects.

### Target Distribution of Potential Effective Constituents in Xiao-Xu-Ming Decoction

Based on the 100 theoretical prediction models which involve 52 targets against AD, the targets of 908 compounds in XXMD were predicted. These 52 targets related to AD referred to cholinergic system dysfunction, glutamate/GABA system dysfunction, aggregates of amyloid-β peptide, hyper-phosphorylated tau, serotonergic system dysfunction, oxidative stress, neuroinflammation, mitochondrial dysfunction, and so on (Table 1). The herb distribution of potential active constituents in XXMD were shown in Figure 1B, from which we can see that more than half of the active constituents derive from *Conioselinum anthriscoides “Chuanxiong”, Glycyrrhiza uralensis Fisch. ex DC., Panax ginseng C.A.Mey*. and *Zingiber officinale Roscoe.*

**TABLE 1:** Classification of Alzheimer's disease (AD) related targets.

Classification of target	Targets
Cholinergic system dysfunction	ACHE, BCHE, CHRM1, CHRM2, CHRNA4, CHRNA7
Glutamate/GABA system dysfunction	GRIA1, GRIA2, GABRG1, GABBR1, GRM2, GRM3, GRIN1
Aggregates of amyloid-β peptide	APP, BACE1, PSEN1
Hyper-phosphorylated tau	HSP90AA1, CDK5, GSK3B, MAPT, PIN1
Serotonergic system dysfunction	HTR1A, HTR2A, HTR3A, HTR4, HTR6
Oxidative stress	MAOB, MPO, PDE4A, PDE4B, PDE9A
Neuroinflammation	MAPK8, MAPK9, MAPK10, MAPKAPK3, CHUK, IKBKB, NOS2, PPARG, TNF, ADORA2A, ALOX12, PTGS2
Mitochondrial dysfunction	PPID, PDHX
Other	ACAT1, COMT, ESR1, HRH3, HMGCS1, IDE, SIGMAR1

**FIGURE 2:**
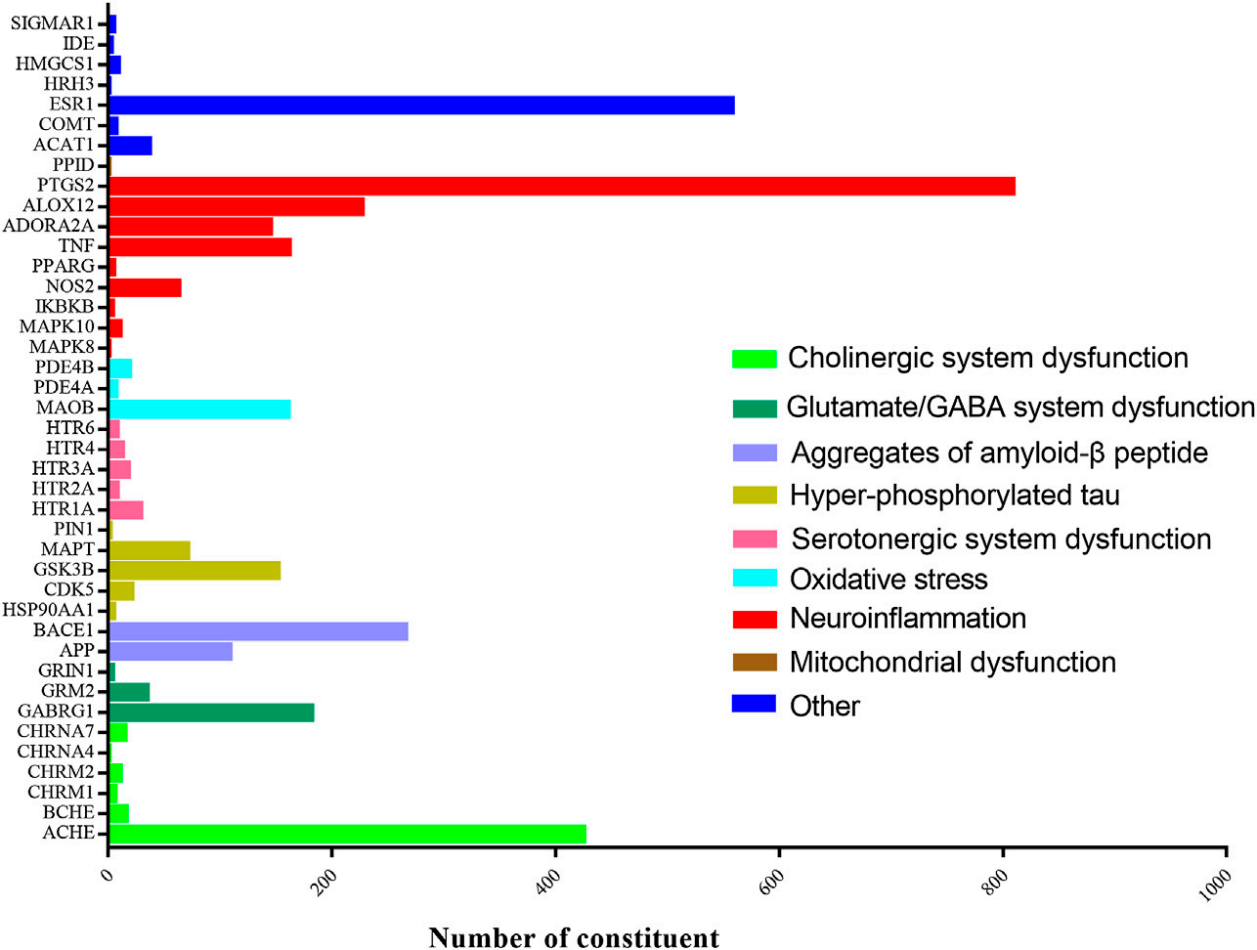
Target distributions of potential active constituent in XXMD.

**FIGURE 3:**
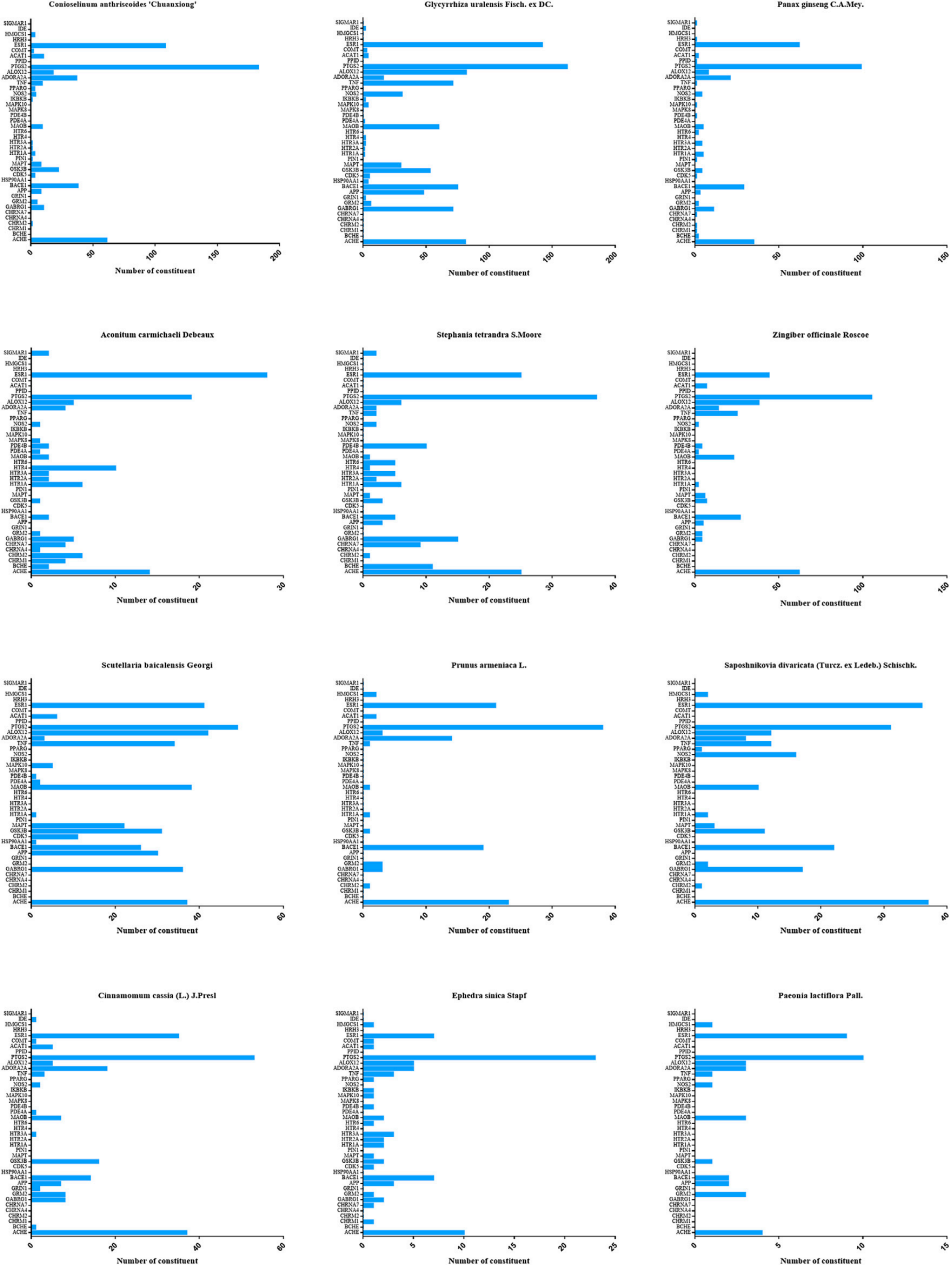
Target distributions of twelve kinds of herbs in XXMD.

To investigate the similarity of twelve herbs in target distribution, the targets prediction for each herb were conducted. As indicated in Figure 2, the constituents in XXMD formula were found to interact with 41 targets associated with AD. As shown in Table 2, cyclooxygenase-2 (COX-2), estrogen receptor α (ERα) and AChE were the major potential targets of constituents in XXMD linked to the treatment of AD. Twelve herbs in the formula were found to interact with the common targets and had similar distribution patterns (Figure 3).

**TABLE 2:** Target distribution of potential active constituents.

No.	Targets	Abbreviation	The number of active constituents
1	cyclooxygenase-2	PTGS2	809
2	estrogen receptor α	ESR1	558
3	acetylcholinesterase	ACHE	425
4	β-secreatase	BACE1	266
5	12-lipoxygenase	ALOX12	227
6	gamma-aminobutyric acid A receptor	GABRG1	182
7	tumor necrosis factor alpha	TNF	162
8	Monoamine oxidase B	MAOB	161
9	glycogen synthase kinase 3 beta	GSK3B	152
10	A2A adenosine receptor	ADORA2A	145
11	beta-amyloid precursor protein	APP	109
12	microtubule-associated protein tau	MAPT	71
13	inducible nitric oxide synthase	NOS2	63
14	Cholesterol Acyltransferase	ACAT1	37
15	metabotropic glutamate receptor 2	GRM2	35
16	5 Hydroxytryptamine 1A receptor	HTR1A	29
17	cyclin-dependent kinase 5	CDK5	21
18	phosphodiesterase type 4B	PDE4B	19
19	5 Hydroxytryptamine 3A receptor	HTR3A	18
20	butyrylcholinesterase	BCHE	16
21	nicotinic acetylcholine receptor α7	CHRNA7	15
22	5 Hydroxytryptamine 4 receptor	HTR4	13
23	c-Jun N-terminal kinase-3	MAPK10	11
24	muscarnic m2 receptor	CHRM2	11
25	3-hydroxy-3-methyl glutaryl coenzyme- A reductase	HMGCS1	9
26	5 Hydroxytryptamine 2A receptor	HTR2A	8
27	5 Hydroxytryptamine 6 receptor	HTR6	8
28	catechol O-methyltransferase	COMT	7
29	phosphodiesterase type 4A	PDE4A	7
30	muscarnic m1 receptor	CHRM1	6
31	heat shock protein 90	HSP90AA1	5
32	Peroxisome proliferator-activated receptor gamma	PPARG	5
33	sigma-1 receptor	SIGMAR1	5
34	nuclear factor kappa-B kinase beta	IKBKB	4
35	N-methyl-D-aspartate receptor	GRIN1	4
36	insulin-degrading enzyme	IDE	3
37	peptidyl prolyl cis/trans Isomerases	PIN1	2
38	c-Jun N-terminal kinase-1	MAPK8	1
39	histamine H3 receptor	HRH3	1
40	nicotinic acetylcholine receptor α4	CHRNA4	1
41	Cyclophilin D	PPID	1

### Network Analysis and Construction

**FIGURE 4:**
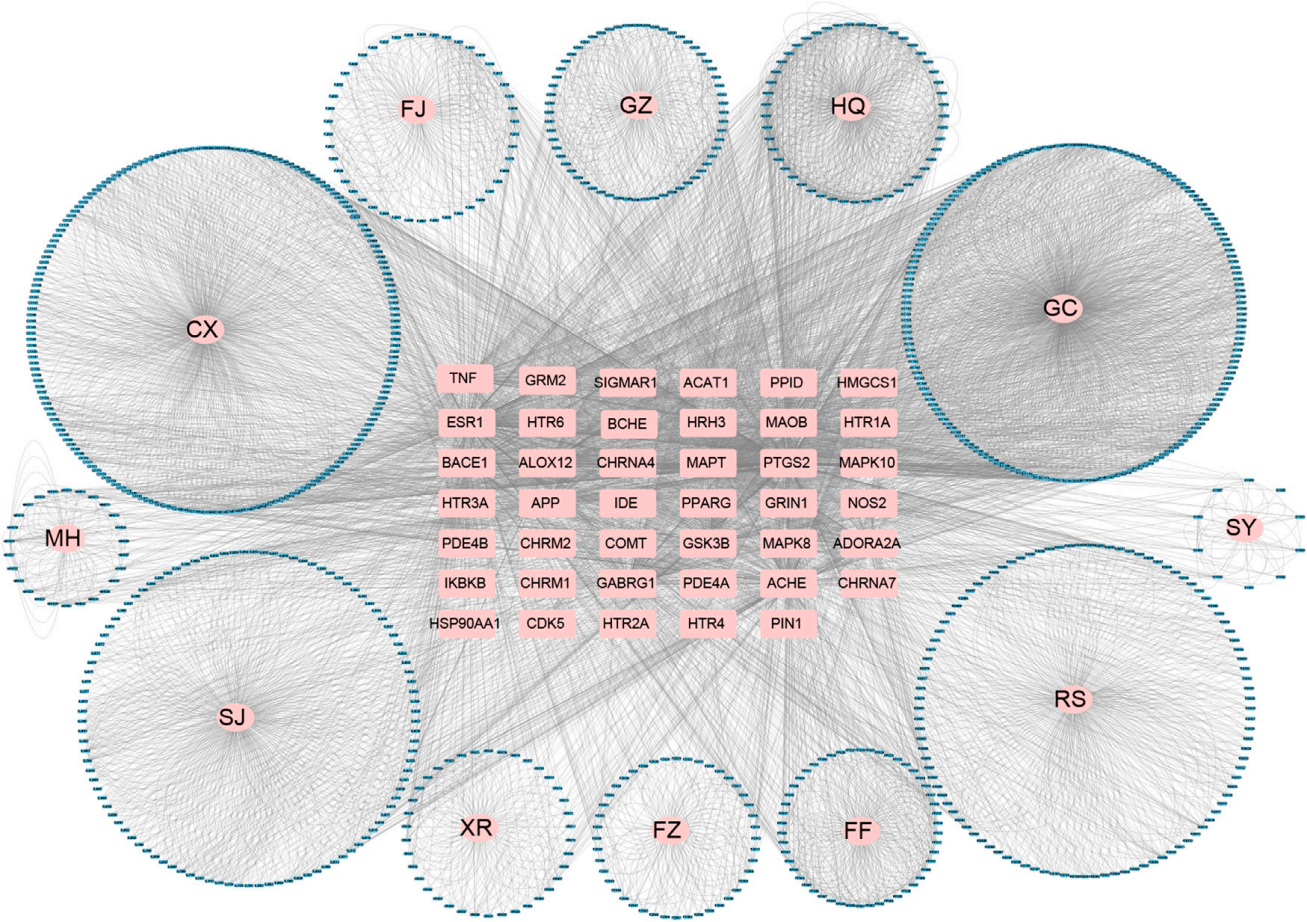
Global constituent-target network of candidate constituents in XXMD. Blue circles correspond to the compounds, pink circles correspond to the source of compounds, and pink rectangles in the center correspond to the target. FF, Fang Feng (*Saposhnikovia divaricata (Turcz. ex Ledeb.) Schischk*.); SJ, Sheng Jiang (*Zingiber officinale Roscoe*); RS, Ren Shen (*Panax ginseng C.A.Mey.*); XR, Xin Ren (*Prunus armeniaca L.*); GC, Gan Cao (*Glycyrrhiza uralensis Fisch. ex DC.*); CX, Chuan Xiong (*Conioselinum anthriscoides 'Chuanxiong'*); MH, Ma Huang (*Ephedra sinica Stapf*); FZ, Fu Zi (*Aconitum carmichaeli Debeaux*); GZ, Gui Zhi (*Cinnamomum cassia (L.) J.Presl*); HQ, Huang Qin (*Scutellaria baicalensis Georgi*); FJ, Fan Ji (*Stephania tetrandra S.Moore*); SY, Shao Yao (*Paeonia lactiflora Pall.*).

To uncover the interactions between the herb constituents and potential targets in treating of AD, the constituent-target (C-T) network based on 908 compounds and potential targets were constructed. 898 of 908 compounds were found to interact with 41 targets associated with AD. As indicated in Figure 4 and Supplementary Table 1, the C-T network consists of 961 nodes (12 herbs, 898 compounds and 41 AD-related targets) and 3632 C-T interactions, resulting in an average degree of 4.04 per compound and 88.58 per target, respectively.

**FIGURE 5:**
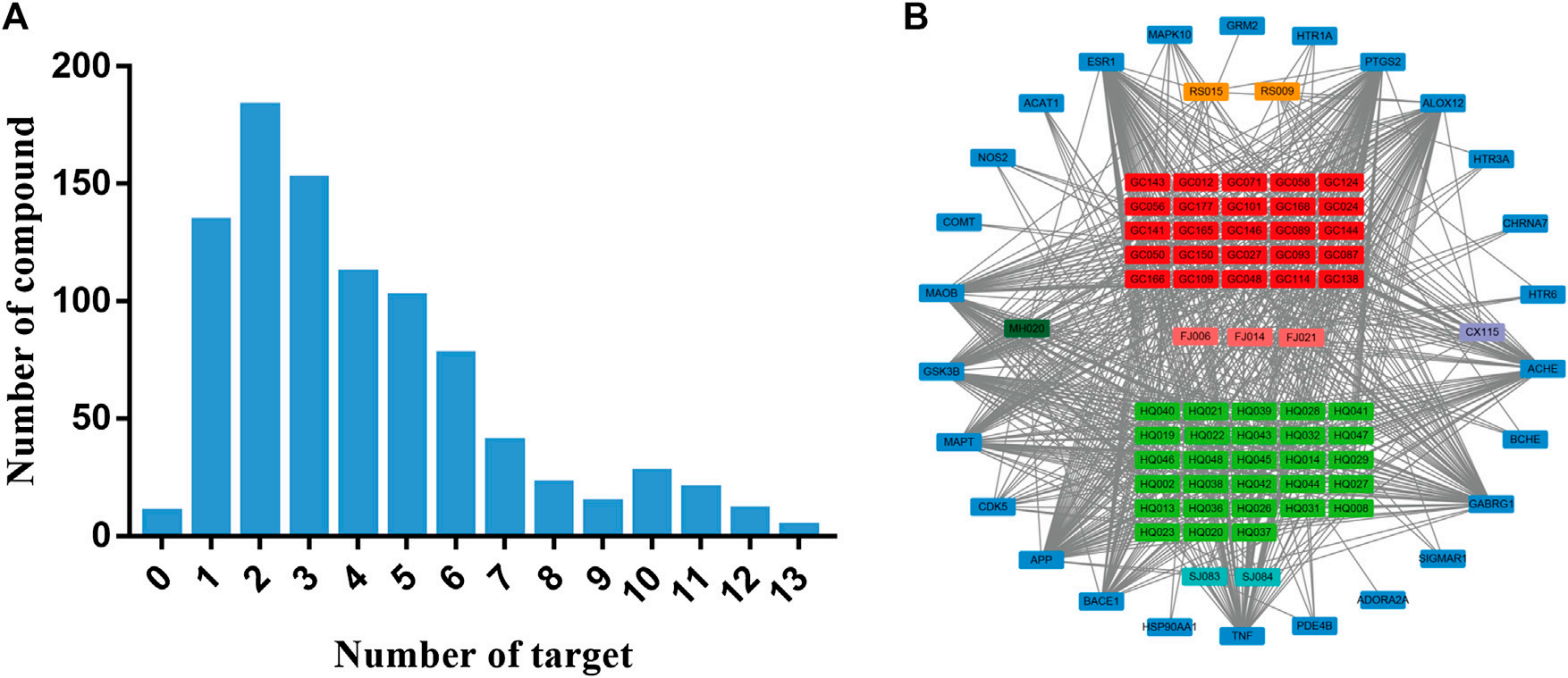
The number of constituent of herbs in XXMD in different frequency of targets **(A)** and constituent-target network for multi-target (≥10) constituent in XXMD **(B)**. In Figure 5B, rectangles in orange, red, purple, green and light blue correspond to the compounds and their sources, and dark blue rectangles correspond to the targets.

According to Figure 5A, most of individual constituents in these 898 compounds were found to interact with multiple AD-related targets (≥2 targets) and the mean number of potential targets per constituent was 4.0. To explore the correlations between the potential active compounds in XXMD and their AD-related targets, for constituents which could interact with more than or equal to 10 targets, we built a constituent-target network. Figure 5B and Supplementary Table 2 indicated that 62 compounds in XXMD were found to interact with more than or equal to 10 targets, and most of them were found to be derived from *Glycyrrhiza uralensis Fisch. ex DC.* and *Scutellaria baicalensis Georgi*. The chemical structures of 62 compounds were shown in Table 3. Among these compounds, 57% of constituents in *Scutellaria baicalensis Georgi* were found to interact with more than or equal to 10 targets, which indicated that *Scutellaria baicalensis Georgi* might be the important herb for XXMD formula treating AD. ESR1 (estrogen receptor α), PTGS2 (cyclooxygenase-2), ALOX12 (12-lipoxygenase), AChE, GABRG1 (gamma-aminobutyric acid A receptor), TNF (tumor necrosis factor alpha), BACE1 (β-secreatase), APP (β-amyloid precursor protein), CDK5 (cyclin-dependent kinase 5), MAPT (microtubule-associated protein tau), GSK3β (glycogen synthase kinase 3 beta) and MAOB (monoamine oxidase B) were hub targets of the network, which suggested that they were main targets for XXMD formula to treat with AD. Thus, most of the active ingredients in XXMD can target multiple targets related to AD.

**TABLE 3:** The chemical structure of constituents binding more than or equal to 10 targets.

No.	Compound	Structure	Source	Number of targets
1	5,7,2'-Trihydroxy-6'-methoxyflavone	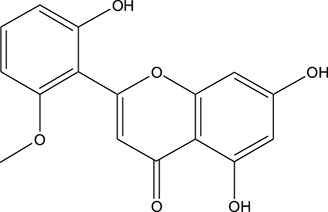	*Scutellaria baicalensis Georgi*	13
2	5,7,4'-Trihydroxy-8-methoxyflavone	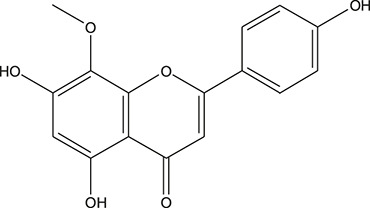	*Scutellaria baicalensis Georgi*	13
3	acacetin	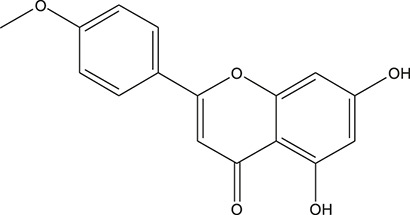	*Scutellaria baicalensis Georgi*	13
4	5-Hydroxy-7,8-dimethoxyflavone	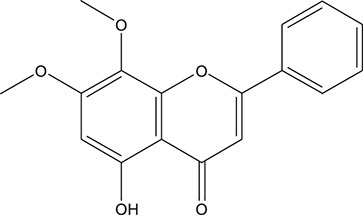	*Scutellaria baicalensis Georgi*	12
5	5,8-Dihydroxy-6,7-dimethoxyflavone	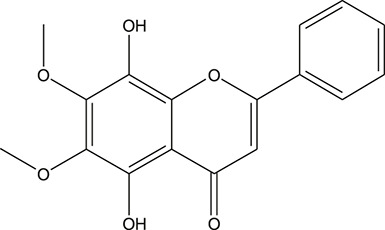	*Scutellaria baicalensis Georgi*	12
6	7-Methoxybaicalein	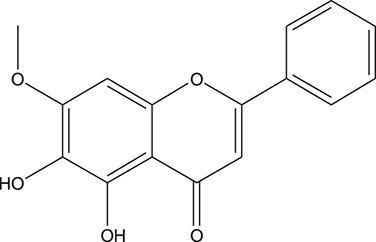	*Scutellaria baicalensis Georgi*	12
7	oroxylin-A	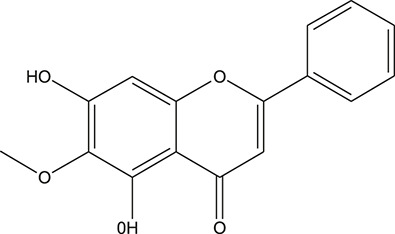	*Scutellaria baicalensis Georgi*	12
8	Salvigenin	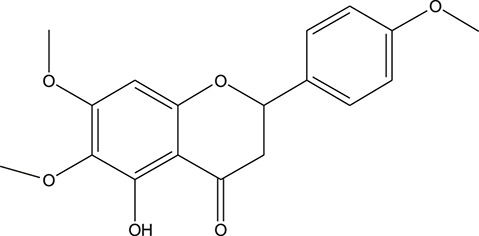	*Scutellaria baicalensis Georgi*	12
9	Wogonin	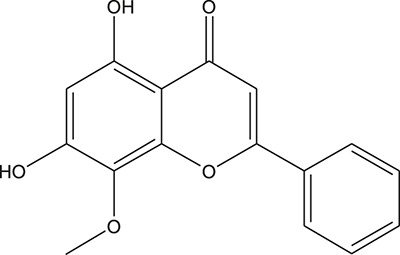	*Scutellaria baicalensis Georgi*	12
10	5,7,2'-Trihydroxy-8-methoxyflavone	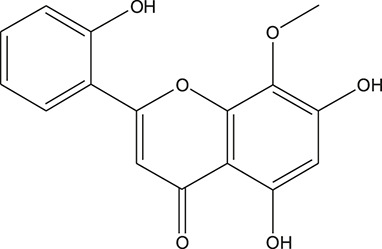	*Scutellaria baicalensis Georgi*	11
11	5, 7, 2'-Trihydroxy-8, 6'-dimethoxyflavone	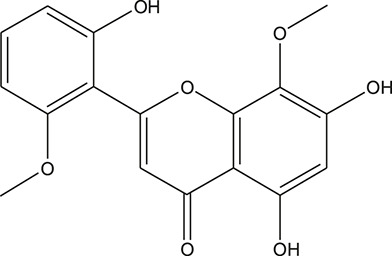	*Scutellaria baicalensis Georgi*	11
12	apigenin	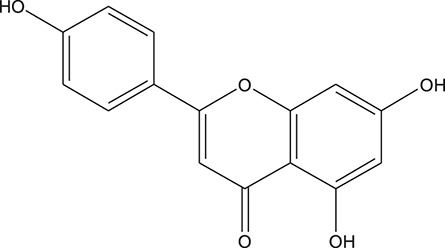	*Scutellaria baicalensis Georgi*	11
13	Baicalein	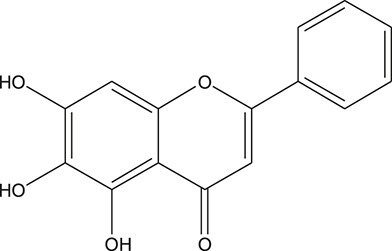	*Scutellaria baicalensis Georgi*	11
14	Chrysin	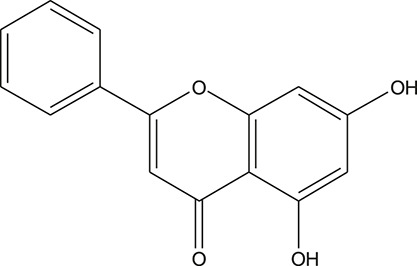	*Scutellaria baicalensis Georgi*	11
15	Neobaicalein	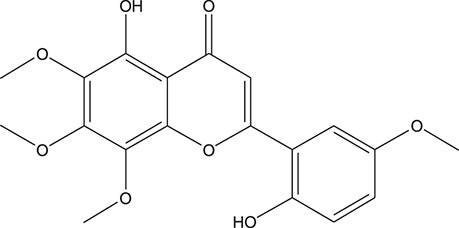	*Scutellaria baicalensis Georgi*	11
16	Norwogonin	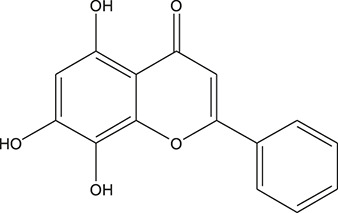	*Scutellaria baicalensis Georgi*	11
17	5,2'-Dihydroxy-6,7,8-trimethoxyflavone	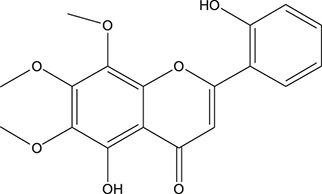	*Scutellaria baicalensis Georgi*	10
18	5,2'-Dihydroxy-6,7,8,6'-tetramethoxyflavone	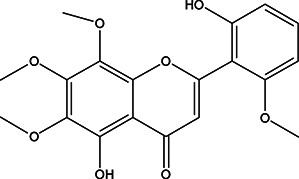	*Scutellaria baicalensis Georgi*	10
19	5,2',6'-Trihydroxy-7,8-dimethoxyflavone	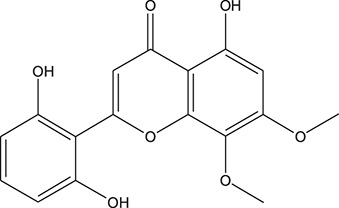	*Scutellaria baicalensis Georgi*	10
20	5,7-Dihydroxy-6,8,2', 3'-tetramethoxyflavone	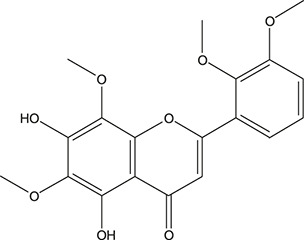	*Scutellaria baicalensis Georgi*	10
21	5, 7, 2'-Trihydroxyflavone	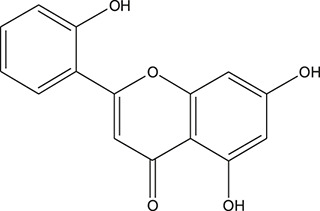	*Scutellaria baicalensis Georgi*	10
22	5, 7, 2'-Trihydroxy-6-methoxyflavone	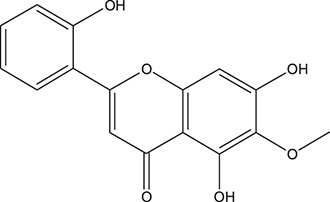	*Scutellaria baicalensis Georgi*	10
23	5,7,2'-Trihydroxy-6, 8-dimethoxyflavone	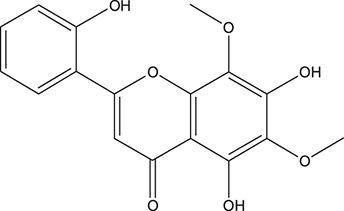	*Scutellaria baicalensis Georgi*	10
24	5, 8, 2'-Trihydroxy-6, 7-dimethoxyflavone	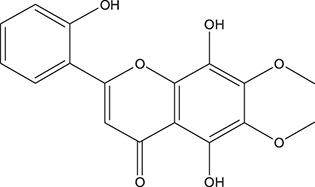	*Scutellaria baicalensis Georgi*	10
25	5,8,2'-Trihydroxy-7-methoxyflavone	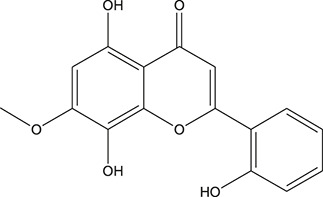	*Scutellaria baicalensis Georgi*	10
26	6,2'-Dihydroxy-5,7,8,6'-tetramethoxyflavone	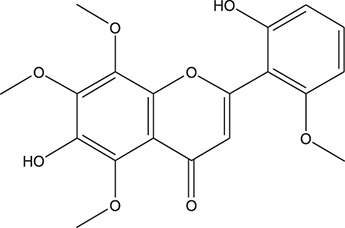	*Scutellaria baicalensis Georgi*	10
27	Oroxylin	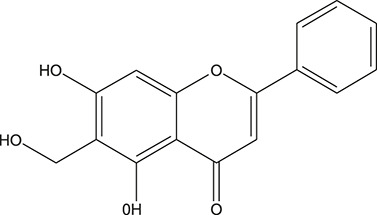	*Scutellaria baicalensis Georgi*	10
28	Panicolin	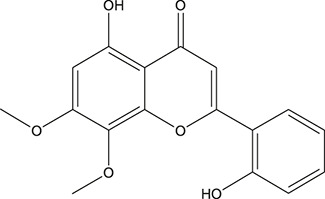	*Scutellaria baicalensis Georgi*	10
29	5,7-dihydroxy-3-(4-methoxyphenyl)-8-(3-methylbut-2-enyl) chromone	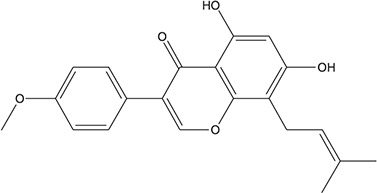	*Glycyrrhiza uralensis Fisch. ex DC.*	12
30	7,2',4'-trihydroxy-5-methoxy-3-arylcoumarin	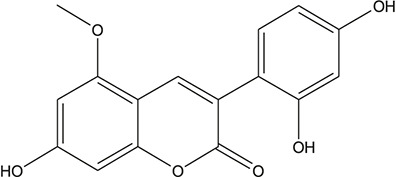	*Glycyrrhiza uralensis Fisch. ex DC.*	12
31	Genkwanin	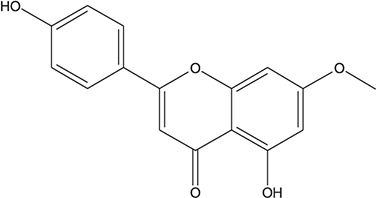	*Glycyrrhiza uralensis Fisch. ex DC.*	12
32	Jaranol	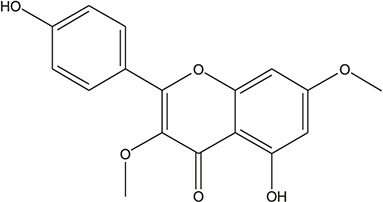	*Glycyrrhiza uralensis Fisch. ex DC.*	12
33	Quercetin der.	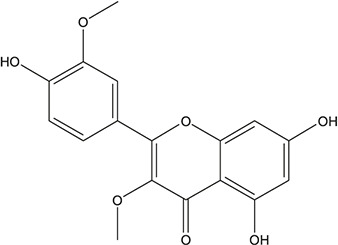	*Glycyrrhiza uralensis Fisch. ex DC.*	12
34	7-Methoxy-4'-hydroxyflavone	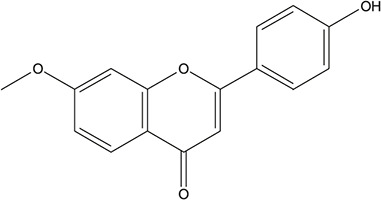	*Glycyrrhiza uralensis Fisch. ex DC.*	11
35	7-Methoxy-4'-hydroxyflavonol	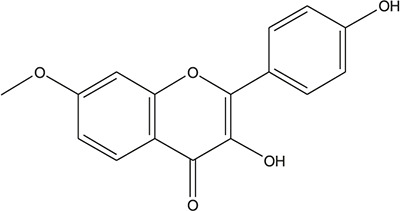	*Glycyrrhiza uralensis Fisch. ex DC.*	11
36	Castanin	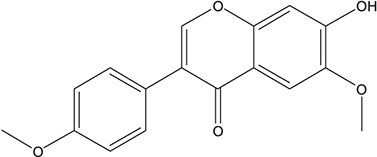	*Glycyrrhiza uralensis Fisch. ex DC.*	11
37	Formononetin	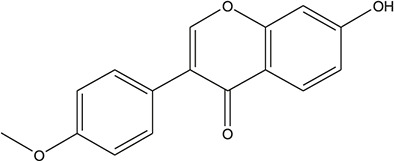	*Glycyrrhiza uralensis Fisch. ex DC.*	11
38	Gancaonin A	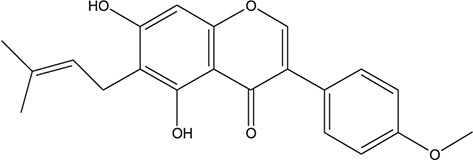	*Glycyrrhiza uralensis Fisch. ex DC.*	11
39	Glypallichalcone	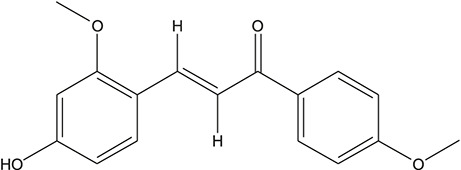	*Glycyrrhiza uralensis Fisch. ex DC.*	11
40	HMO	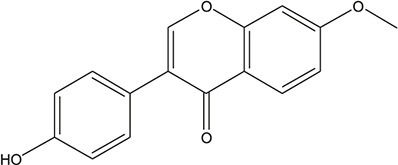	*Glycyrrhiza uralensis Fisch. ex DC.*	11
41	Pallidiflorin	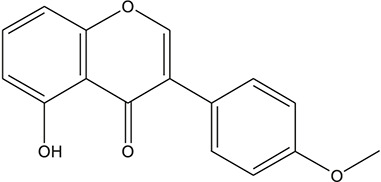	*Glycyrrhiza uralensis Fisch. ex DC.*	11
42	Prunetin	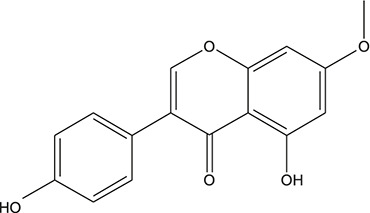	*Glycyrrhiza uralensis Fisch. ex DC.*	11
43	Yinyanghuo D	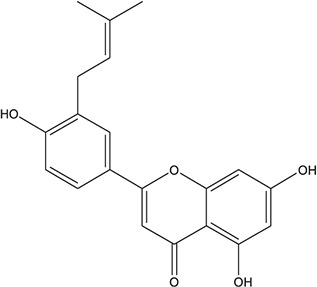	*Glycyrrhiza uralensis Fisch. ex DC.*	11
44	1,3-dihydroxy-9-methoxy-6-benzofurano[3,2-c] chromenone	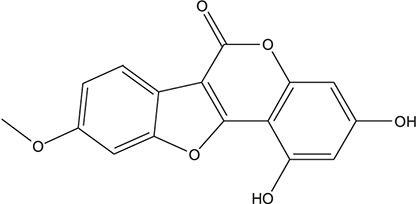	*Glycyrrhiza uralensis Fisch. ex DC.*	10
45	3,4,3',4'-Tetrahydroxy-2-methoxychalcone	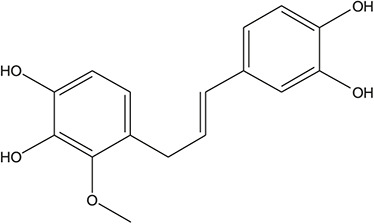	*Glycyrrhiza uralensis Fisch. ex DC.*	10
46	4'-methoxyflavone	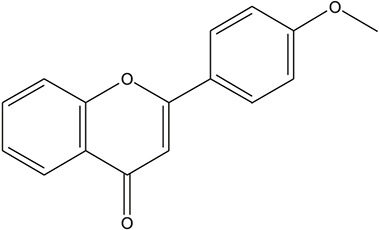	*Glycyrrhiza uralensis Fisch. ex DC.*	10
47	Calycosin	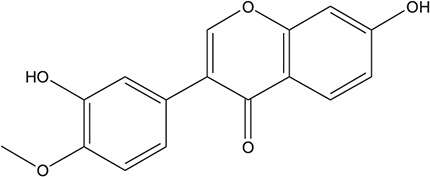	*Glycyrrhiza uralensis Fisch. ex DC.*	10
48	Daidzein dimethyl ether	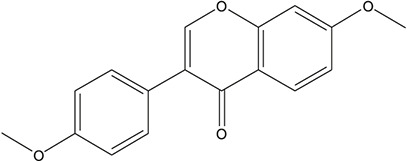	*Glycyrrhiza uralensis Fisch. ex DC.*	10
49	Gancaonin G	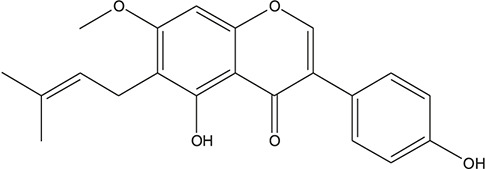	*Glycyrrhiza uralensis Fisch. ex DC.*	10
50	Karenzu DK2	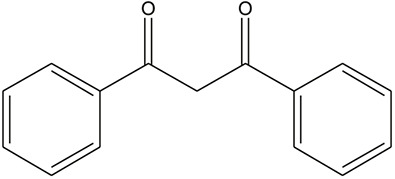	*Glycyrrhiza uralensis Fisch. ex DC.*	10
51	Licoricone	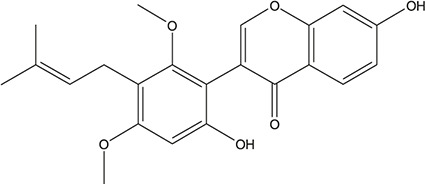	*Glycyrrhiza uralensis Fisch. ex DC.*	10
52	Licochalcone B	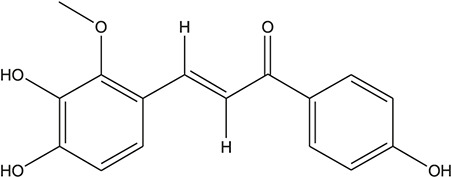	*Glycyrrhiza uralensis Fisch. ex DC.*	10
53	Odoratin	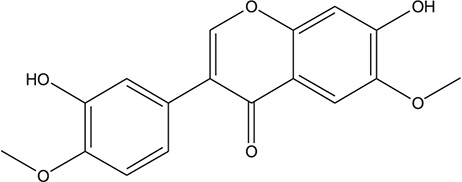	*Glycyrrhiza uralensis Fisch. ex DC.*	10
54	tricin	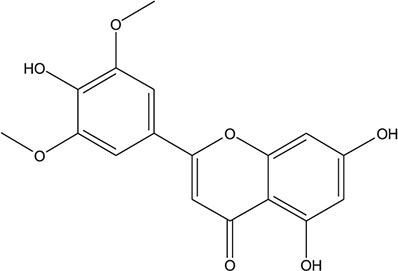	*Ephedra sinica Stapf*	13
55	fangchinoline	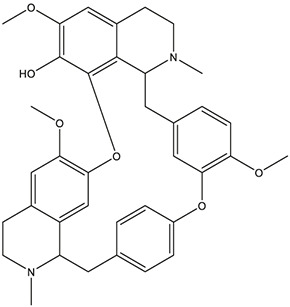	*Stephania tetrandra S.Moore*	11
56	Curine	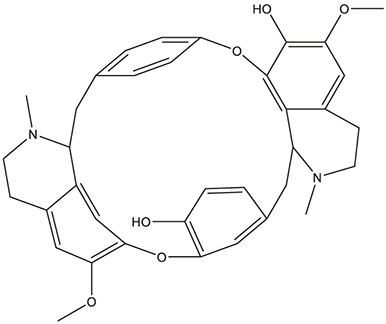	*Stephania tetrandra S.Moore*	10
57	TNP00326	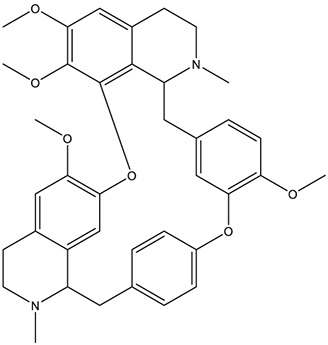	*Stephania tetrandra S.Moore*	10
58	Dauricine (8CI)	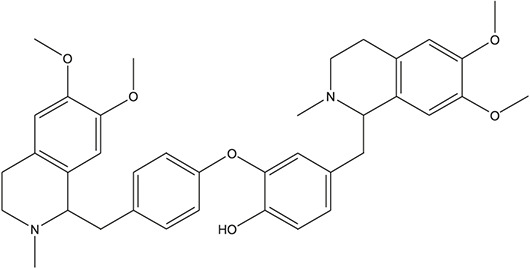	*Panax ginseng C.A.Mey.*	11
59	kaempferol	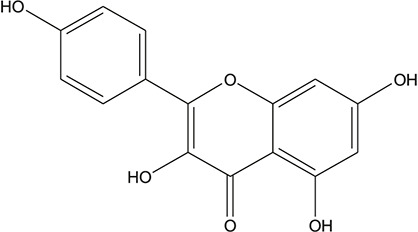	*Panax ginseng C.A.Mey.*	11
60	Coniferylfcrulate	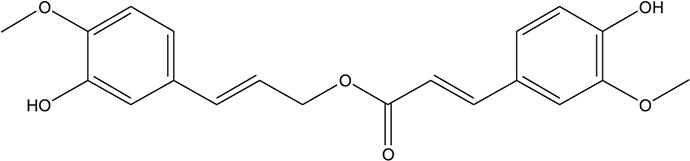	*Conioselinum anthriscoides 'Chuanxiong'*	10
61	Gingerenone A	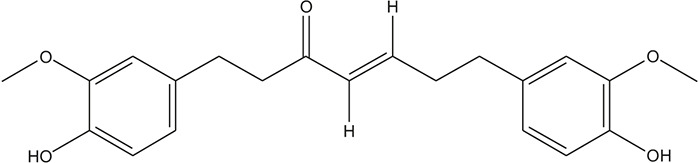	*Zingiber officinale Roscoe*	10
62	Gingerenone C	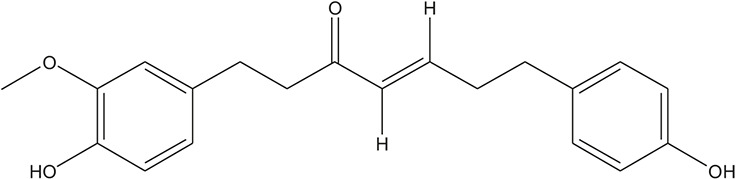	*Zingiber officinale Roscoe*	10

**FIGURE 6:**
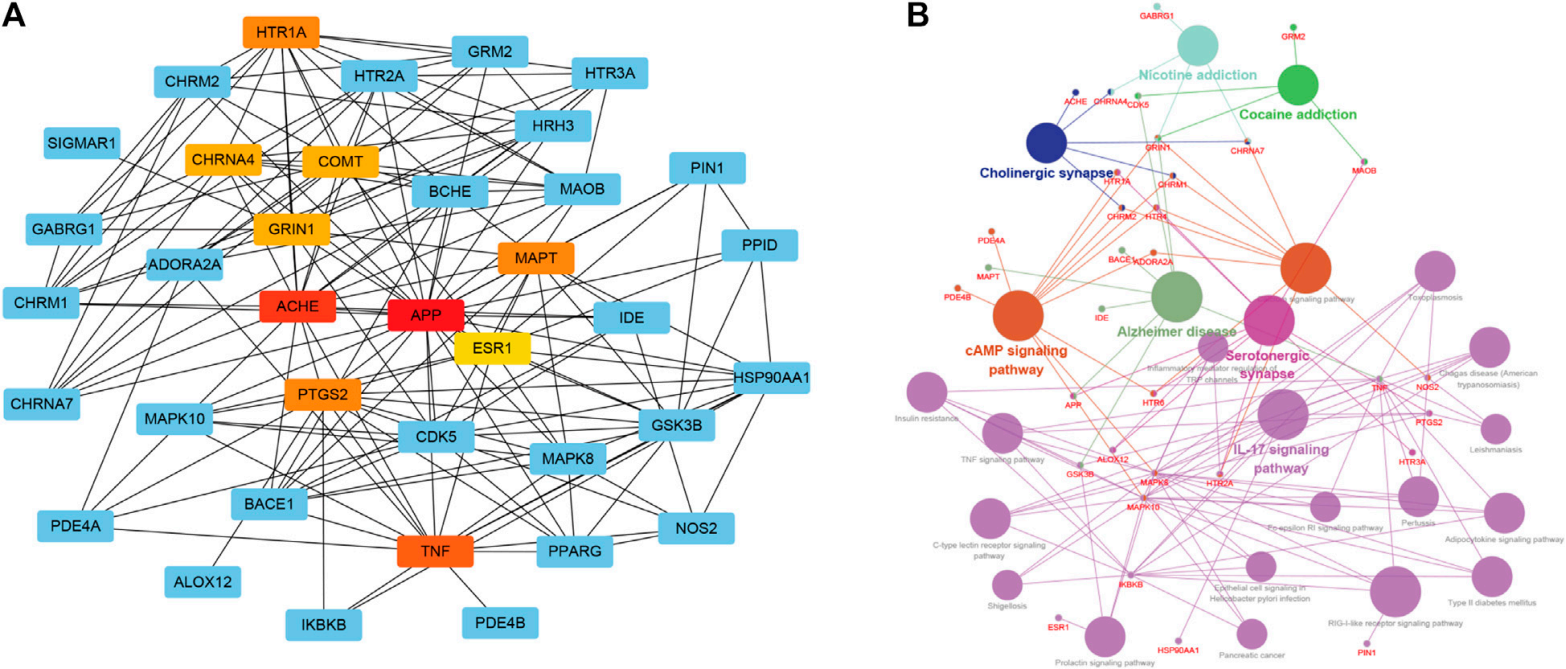
Target-target network **(A)** and target-function network **(B)** of candidate constituents in XXMD. A functional module is linked to a target if the target is involved in that biological process. In Figure 6B, red words correspond to the targets, and black words correspond to the AD-related functional modules.

To furtherly establish the correlations among the XXMD associated AD targets, a “target-target network” was built based on the STRING results of the interactions among 41 AD-related targets (Figure 6A). As shown in target−target network, AChE, APP, TNF, PTGS2, HTR1A and MAPT were high-latitude nodes (Hub), and these hub nodes exerted important roles in the treatment of AD. The top 10 hub genes with higher degree of connectivity were shown in Table 4.

**TABLE 4:** Top 10 hub genes with higher degree of connectivity.

Rank	Target	Score
1	APP	26
2	ACHE	17
3	TNF	15
4	PTGS2	13
5	HTR1A	13
6	MAPT	13
7	GRIN1	12
8	CHRNA4	12
9	COMT	12
10	ESR1	11

After network analysis, the targets were further mapped into GO and KEGG database for extracting the pathways which were closely linked with these targets. The target-function network for potential inhibitors was shown in Figure 6B, this network consists of 33 targets with 24 AD-related functional modules. These modules consist of cAMP signaling pathway, serotonergic synapse, IL-17 signaling pathway, cholinergic synapse, TNF signaling pathway, type II diabetes mellitus and so on.

### Inhibitory Activity of Active Constituents in Xiao-Xu-Ming Decoction on acetylcholinesterase

**FIGURE 7:**
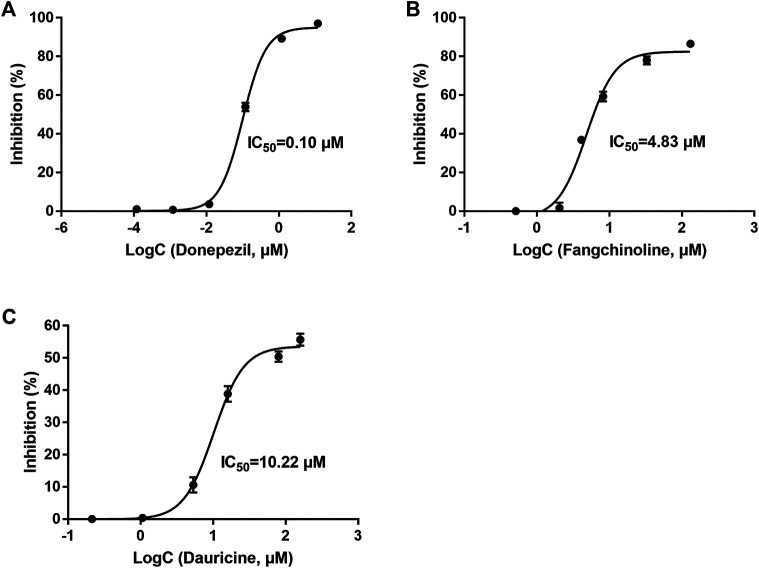
The inhibitory effect of Donepezil **(A)**, Fangchinoline **(B)** and Dauricine **(C)** on acetylcholinesterase activity. Results are presented as means ± SEM, *n* = 3.

AChE is one of the major targets of constituents in XXMD on the treatment of AD, and it is also regarded as one of the drug targets for the treatment of AD. Hence, available 7 compounds interacting with more than 10 AD-related targets (Table 5) were further evaluated regarding inhibitory activity of AChE by *in vitro* assays. At the concentration of 5 μg/ml, fangchinoline and dauricine were found to display significant AChE inhibitory activity (72.13% and 42.05%, respectively), compared to that of the reference compound donepezil (97.03%) which was used as the reference compound. Baicalein, chrysin, oroxylin A, quercetin and wogonin had weak inhibitory activity on AChE (Table 5). Donepezil was with an IC_50_ value of 0.10 μM. Among the seven constituents, two constituents of fangchinoline and dauricine were identified to exhibit moderate inhibitory activity toward AChE, with the IC_50_ values as 4.83 μM and 10.22 μM, respectively (Figure 7). Fangchinoline (IC_50_ = 4.83 μM) was the most active compound in 7 compounds interacting with more than 10 AD-related targets and deserved the further study.

**TABLE 5:** The detailed information of compounds from Xiao-Xu-Ming Decoction.

Compound	Targets	Inhibition (%) against AChE	CDOCKER interaction energy of compounds interacting with AChE
Baicalein	ALOX12, ACAT1, APP, BACE1, CDK5, PTGS2, ESR1, GABRG1, GSK3B, MAOB, TNF	3.35	41.0117
Chrysin	ALOX12, APP, BACE1, CDK5, PTGS2, ESR1, GABRG1, GSK3B, MAPK10, MAOB, TNF	−1.31	40.5337
Dauricine	ALOX12, HTR1A, HTR3A, HTR6, ACHE, APP, BCHE, PTGS2, GABRG1, PDE4B, SIGMAR1	42.06	84.4849
Fangchinoline	HTR1A, HTR3A, HTR6, ACHE, CHRNA7, APP, BCHE, PTGS2, GABRG1, PDE4B, SIGMAR1	72.13	55.9794
Oroxylin A	ALOX12, ACHE, APP, BACE1, CDK5, PTGS2, ESR1, GABRG1, GSK3B, MAOB, MAPT, TNF	−2.99	46.1022
Quercetin	ALOX12, ACHE, APP, CDK5, PTGS2, ESR1, GABRG1, GSK3B, MAPK10, MAOB, MAPT, TNF	−4.96	43.9071
Wogonin	ALOX12, ACHE, APP, BACE1, CDK5, PTGS2, ESR1, GABRG, GSK3B, MAOB, MAPT, TNF	7.98	41.485

### Target Identification of Fangchinoline and Dauricine

Based on the prediction results of machine learning, fangchinoline was found to interact with 11 AD-related targets (ACHE, BCHE, APP, PTGS2, PDE4B, CHRNA7, GABRG1, SIGMAR1, HTR1A, HTR3A and HTR6) and dauricine was also found to interact with 11 AD-related targets (ACHE, BCHE, APP, PTGS2, PDE4B, ALOX12, GABRG1, SIGMAR1, HTR1A, HTR3A and HTR6) (Table 5).

**FIGURE 8:**
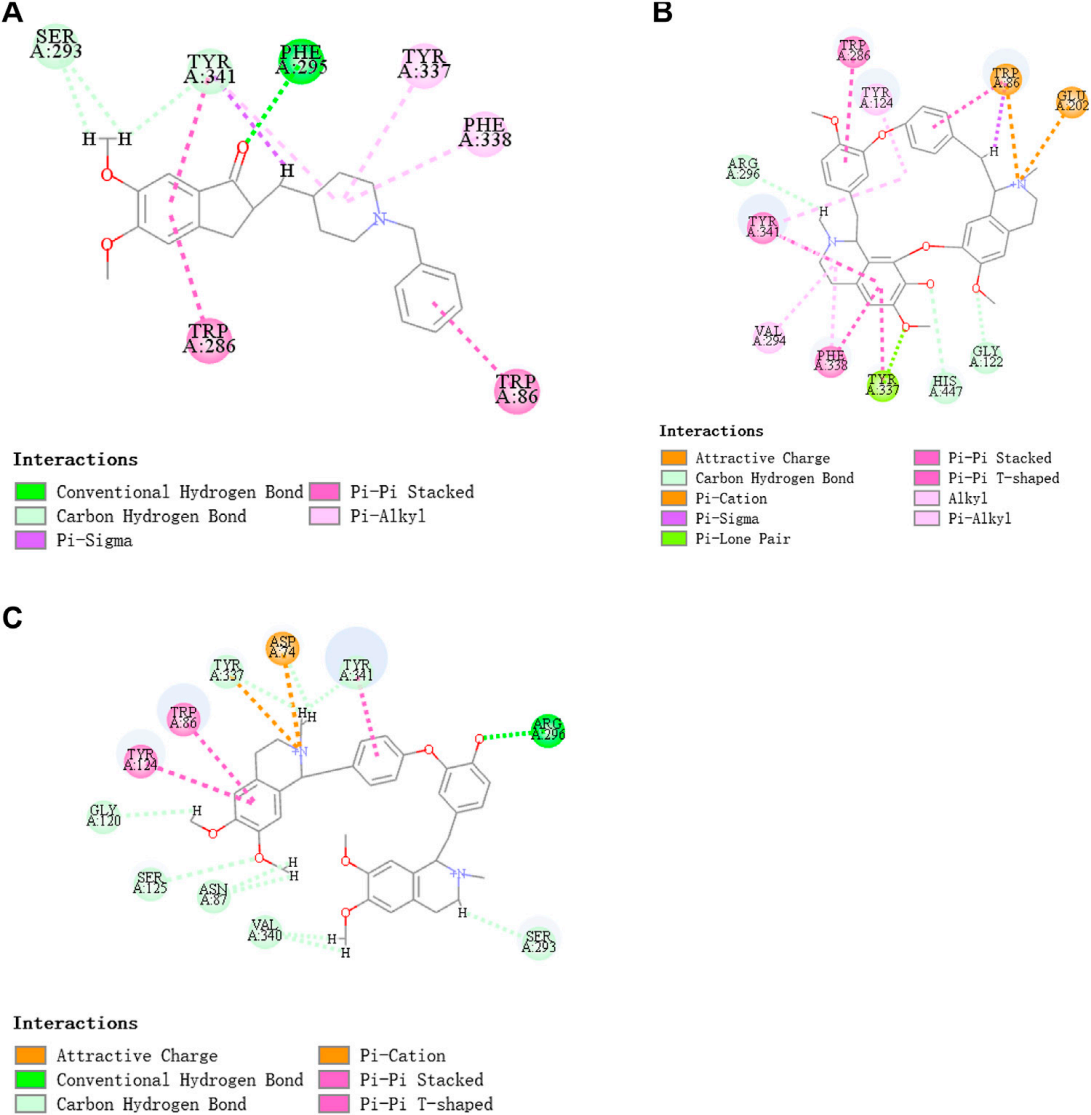
Donepezil **(A)**, Fangchinoline **(B)** and Dauricine **(C)** interacting with acetylcholinesterase (AChE), the AChE model was established by molecular docking.

As *in vitro* AChE inhibitory assay results illustrated that fangchinoline and dauricine exhibited moderate activities against AChE, molecular docking was then used to identify the interaction between the compounds and AChE. The ligand donepezil was extracted from the AChE crystal structure (PDB ID: 4EY7), and then re-docked into the active site of AChE. The root-mean-square distance (RMSD) values of the docked poses in the crystallographic complex were 0.1939, indicating the high accuracy and reliability of the docking methods. Figure 8 showed that fangchinoline and dauricine could interact with AChE, with CDOCKER interaction energy as 55.9794 and 84.4849, respectively. Donepezil was used as the reference compound with CDOCKER interaction energy of 56.9993. The interaction of donepezil and AChE was shown in Figure 8A, while the head group amide substituent of donepezil formed conventional hydrogen bond with amino acids Phe295. Other interactions of donepezil included Pi-Pi stacked interactions with Trp286, Trp86 and Tyr341 and Pi-alkyl interactions with Tyr341, Tyr337, Phe338. The donepezil is stacked with Trp286 of AChE in the peripheral anionic site, and the benzyl ring is stacked with Trp86 in the catalytic anionic site of the enzyme. As indicated in Figure 8B, fangchinoline has the similar interaction mode with donepezil. Fangchinoline formed Pi-Pi stacked interactions with Trp286, Trp86, Tyr341, Phe338 and Pi-alkyl interactions with Tyr341, Tyr124, Phe338 and Val294. Dauricine was found to interact with Asp296 via one conventional hydrogen bond, and Pi-Pi stacked interactions with Trp86, Tyr124 and Tyr341, as well as Tyr337 and Asp74 via Pi-cation (Figure 8C). The CDOCKER interaction energy of the available 7 compounds interacting with more than 10 AD-related targets were shown in Table 5. The results showed that the CDOCKER interaction energy of fangchinoline and dauricine were greater than those of baicalein, chrysin, oroxylin A, quercetin and wogonin.

### Neuroprotective Effect of Fangchinoline

**FIGURE 9:**
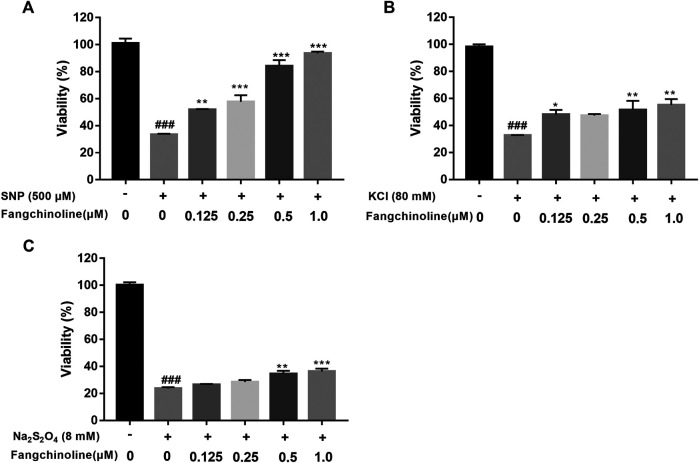
The protective effects of fangchinoline against cell injury induced by sodium nitroprusside **(A)**, potassium chloride **(B)** and sodium dithionate **(C)** in SH-SY5Y cells. Results are presented as means ± SEM, *n* = 3. ### *P*<0.001 versus each control group. * *P*<0.05, ** *P*<0.01, *** *P*<0.001 versus group solely treated with sodium nitroprusside, potassium chloride or sodium dithionate in **(A)**, **(B)** and **(C)**, respectively.

As fangchinoline was the most active compound with AChE inhibitory activity among the available 7 compounds obtained from XXMD database, it was then chosen for the further *in vitro* cell experiments to assess its actual neuroprotective effects. Compared with the control group, the viability of cells pre-incubated with fangchinoline was reduced while under the sodium nitroprusside, sodium dithionate or potassium chloride treatment. Cell viability was significantly improved in the groups pre-treated with 0.25 μM, 0.5 μM, and 1 μM fangchinoline than in the group treated with sodium nitroprusside, sodium dithionate and potassium chloride alone (Figure 9). Moreover, the neuroprotective effect of fangchinoline on SH-SY5Y cells against sodium nitroprusside induced toxicity was more obvious than that in the sodium dithionate or potassium chloride induced cell injured models.

## Discussion

Network Pharmacology has become a promising approach in current drug discovery and development, especially in TCM research. Unlike “one gene, one target, one disease” strategy, network pharmacology affords a novel network mode of “multiple targets, multiple effects, complex diseases” and coincides with the characteristics of TCM and holistic view of TCM treatment (Zhang et al., 2013). To date, it remains a challenge for researchers to discover satisfactory drug for AD in modern medicine. Increasing evidence suggested that traditional Chinese medicine could be applied to the prevention and treatment of AD (Fang et al., 2017b; Pang et al., 2018). Our study aimed to evaluate the mechanisms of the constituents in XXMD for the potential treatment of AD. In this study, network pharmacology method which integrated drug-likeness filtering, target prediction and network analysis was used to dissect the potential targets and the material basis of XXMD for the potential AD treatment.

According to the target distribution of potential effective constituents in XXMD, the inflammatory reaction and cholinergic system were the main pathways which XXMD affect, and COX-2, ERα and AChE were the major targets of constituents in XXMD linked to the potential treatment of AD. Among them, COX-2 and ERα are not the reported specific drug targets for AD. COX-2 plays a vital role in inflammatory reactions and has emerged as a major player in neurological diseases such as multiple sclerosis, amyotrophic lateral sclerosis, Parkinson disease and AD (Minghetti, 2004). Estrogen is one of transcription factors which exert neuroprotective activity in pathological process of AD, such as synaptotoxicity, neuroinflammation, oxidative stress, Aβ accumulation, tau phosphorylation and mitochondrial bioenergetics (Merlo et al., 2017). Estrogen response is mainly mediated by estrogen receptor α (ER α) and estrogen receptor β (ER β). Previous studies found that ERα co-localized with neurofibrillary pathology and interacted with tau protein in AD brain (Wang et al., 2016). While AChE is the key enzyme in the hydrolysis of the neurotransmitter acetylcholine (Saxena and Dubey, 2019). The cholinergic hypothesis suggests that AD arises due to the dysfunction of acetylcholine containing neurons in the brain and most of the clinically used anti-AD drugs preserve acetylcholine inhibiting AChE. Thus, it will likely remain pivotal for rational drug development for the treatment of AD to target acetylcholine deficiency.

Therefore, available 7 compounds interacting with more than 10 AD-related targets in XXMD were furtherly subjected to the *in vitro* AChE Inhibitory Assay to assess their inhibitory activity of AChE. Our data showed that among these constituents, fangchinoline and dauricine had potent inhibitory effects on the activity of AChE. These results suggest that fangchinoline and dauricine might be the potential constituents in XXMD for the potential AD treatment. Dauricine is a benzyl tetrahydroisoquinoline alkaloid isolated from the root of *Menispermum dauricum DC.,* and it has been found to have significant neuroprotective effect on AD. Dauricine has the pharmacological activity of inhibiting APP processing, reducing Aβ accumulation, attenuating the hyperphosphorylation of tau (Liu et al., 2018), anti-oxidative and anti-apoptosis, and shows the potential therapeutic value for AD (Wang et al., 2020).

Fangchinoline is derived from *Stephania tetrandra S.Moore* and has wide range of pharmacological effects. Many studies have showed that fangchinoline had neuroprotective effects by inhibiting oxidative neuronal damage induced by glutamate (Bao et al., 2019). Our study identified the potential inhibitory activity of fangchinoline on AChE, and it could prevent SH-SY5Y cells from the cytotoxicities induced by sodium nitroprusside, sodium dithionate and potassium chloride. However, there is no other report available for the therapeutic effect of fangchinoline on AD. Hence, it deserved further research.

Among 62 constituents in XXMD interacting with more than or equal to 10 AD-related targets, most of them were found to be derived from *Glycyrrhiza uralensis Fisch. ex DC.* and *Scutellaria baicalensis Georgi*. Fangchinoline and dauricine, which had moderate activities against AChE, were obtained from *Stephania tetrandra S.Moore* and *Panax ginseng C.A.Mey.,* respectively. The results maybe due to other constituents had no significant inhibitory activity against AChE. Wogonin, one of the major active constituents in *Scutellaria baicalensis Georgi,* has been reported to had protective effects against AD by inhibition of amyloidogenic pathway (Huang et al., 2017) and tau phosphorylation (Zhu and Wang, 2015). The flavonoid baicalein, which is derived from *Scutellaria baicalensis Georgi*, is reported to have the ability of inhibiting GSK3β activity, reducing β-secretase enzyme (BACE1), decreasing the concentration of total Aβ, and preventing phosphorylation of tau in APP/PS1 mice (Gu Gu X.-H. et al., 2016). Quercetin is derived from *Glycyrrhiza uralensis Fisch. ex DC.,* and has been shown to protect neurons from oxidative damage, inhibit the fibril formation of amyloid-proteins and inflammatory cascade pathways in AD (Khan et al., 2019).

To evaluate the neuroprotective effects of fangchinoline during the pathology progression of AD, three cell damaged models were established. As the NO donor, Sodium nitroprusside (SNP) causes neural damage. NO is a small gas molecule that can permeate the cell membrane and directly modifies its intracellular targets. Previous studies have shown that NO regulates intracellular calcium channels by interacting with cyclic GMP (cGMP) and promotes the formation of reactive nitrogen (RNS) and oxidative damage through interacting with ROS. NO regulates the release of proinflammatory molecules and targets vital organelles such as mitochondria, ultimately leading to the death of nerve cells, a hallmark of many neurodegenerative diseases including AD (Asiimwe et al., 2016). Sodium dithionite (Na2S2O4) was applied to cause chemical hypoxia that simulates the hypoxic condition in the pathogenesis of AD (Lin et al., 2014). Potassium chloride causes depolarization of neuronal cells and promotes voltage-dependent calcium channels opening, leading to increases in intracellular calcium ion levels. Overloading of calcium ions in neurons can cause neuronal apoptosis (Ishikawa et al., 2017). The validation of fangchinoline against three cell damaged models also supported its potential usage in AD therapy. Therefore, our results suggest that the effect of fangchinoline on AD deserves further study.

In conclusion, XXMD could interact with 41 targets associated with AD and the constituents in XXMD were found to be linked to the potential AD treatment through multiple AD-related targets. 62 constituents in XXMD were found to interact with more than or equal to 10 AD-related targets, among them, fangchinoline and dauricine might be the potential lead compounds in XXMD for the treatment of AD. We were the first to elucidate the material basis of action for XXMD for AD treatment, using the virtual screening and network pharmacology method. Our study first identified the potential activity of fangchinoline against AD. The study was expected to broaden the options of AD treatment methods and further demonstrate the feasibility to apply network pharmacology to the analysis of TCM prescriptions. Still, further *in vivo* experiment needs to be done to verify the effects of XXMD and its constituents such as fangchinoline on AD. Moreover, just two compounds (fangchinoline and dauricine) were determined as the potential active ingredient in XXMD against AD, more experiments need to be done to screen other potential constituents with anti-AD activity, especially those constituents interacting with more than or equal to 10 targets.

## Data Availability Statement

The original contributions presented in the study are included in this article, further inquiries can be directed to the corresponding authors.

## Ethics Statement

The animal study was reviewed and approved by The Animal Care &Welfare Committee of Institute of Materia Medica, CAMS&PUMC.

## Author Contributions

YS and LL designed the study. YS, BZ, XP, RY, MC, JZ, ZW, and ZY performed the experiments. YS and BZ analyzed the data. LL and GD supervised the study. YS drafted the text. JW, YW, LL, AL, and GD corrected the manuscript.

## Funding

This work was supported by grants from CAMS Innovation Fund for Medical Sciences (CIFMS) (2016-I2M-3-007), Major Scientific and Technological Special Project for “Significant New Drug Creation” (2013ZX09508104, 2013ZX09402203, 2018ZX09711001-003-005, 2018ZX09711001-001-015), the China Scholarship Council (201808110107), National Natural Science Foundation of China (81473383) and the Beijing National Science Foundation (7192134).

## Conflict of Interest

The authors declare that the research was conducted in the absence of any commerical or financial relationships that could be construed as a potential conflict of interest.
