# Supplementary material for: Network Pharmacology-Based Analysis of Xiao-Xu-Ming Decoction on the Treatment of Alzheimer’s Disease
Source: Front Pharmacol. 2020 Dec 4;11:595254. doi: 10.3389/fphar.2020.595254 (PMC7774966; doi:10.3389/fphar.2020.595254)
Supplement: Supplementary file 1 [file Table1_v1.DOCX]

**Cover letter**

Dear editor,

We would like to submit the enclosed manuscript entitled “Network Pharmacology-based analysis of Xiao-Xu-Ming Decoction on the Treatment of Alzheimer's Disease” to be considered for publication in the special issue named “Integrative Pharmacology-based Research on Traditional Medicine: Methodologies, Medical and Pharmacological Applications” in Frontiers in Pharmacology as *Research Paper*.

Xiao-Xu-Ming Decoction (XXMD) has been widely used to treat stroke and other neurological diseases for more than 1000 years in China. Learning and memory improvement ability of XXMD has also been reported in rats with chronic cerebral ischemia or aging. However, the synergistic mechanism of the constituents in XXMD for the potential treatment of AD still remains unclear. Network pharmacology has great potentials in identifying alternative targets for herbal medicines and developing multi-target drugs. It has provided new insight for us to study traditional Chinese Medicine (TCM). In this manuscript, we present evidence that the constituents in XXMD formula could interact with 41 targets associated with AD, and cyclooxygenase-2 (COX-2), estrogen receptor α (ER) and acetylcholinesterase (AChE) were the major targets. The constituents in XXMD were found to be linked to the potential AD treatment through multiple AD-related targets. 62 constituents in XXMD were found to interact with more than or equal to ten AD-related targets. Among them, fangchinoline and dauricine might be the potential lead compounds in XXMD for the treatment of AD. To the best of our knowledge, we were the first to elucidate the mechanisms of action for XXMD for AD treatment, using the virtual screening and network pharmacology method. Our study first identified the potential activity of fangchinoline against AD. Our work demonstrates the feasibility to apply network pharmacology to analyze TCM prescriptions and implicates the therapeutic effects of XXMD on AD. Therefore we would like to present our exciting discovery to the readers of Frontiers in Pharmacology. Your prompt consideration of our manuscript would be greatly appreciated.

The manuscript, or part of it, has neither been published nor is currently under consideration for publication by any other journal. All of the co-authors have read the manuscript and approved its submission to Frontiers in Pharmacology.

Sincerely Yours

Li Li, PhD, Associate Professor of Pharmacology

Guan-Hua Du, PhD, Professor of Pharmacology

Beijing Key Laboratory of Drug Target and Screening Research

Institute of Materia Medica

Chinese Academy of Medical Sciences and Peking Union Medical College

No.1 Xian Nong Tan Street

Beijing 100050, P.R. China.

Tel/Fax: 86-10-63165184

Email: lili@imm.ac.cn

Email: dugh@imm.ac.cn
