# Supplementary material for: Network Pharmacology-Based Analysis of Xiao-Xu-Ming Decoction on the Treatment of Alzheimer’s Disease
Source: Front Pharmacol. 2020 Dec 4;11:595254. doi: 10.3389/fphar.2020.595254 (PMC7774966; doi:10.3389/fphar.2020.595254)
Supplement: Supplementary file 2 [file Table2_v1.DOC]

Dear editors:

Thank you very much for all of your hard work and constructive suggestions on our manuscript entitled “Network Pharmacology-based Analysis of Xiao-Xu-Ming Decoction on the Treatment of Alzheimer's Disease” (Manuscript Number: 595254) which we really appreciated. We now would like to re-submit the newly revised manuscript. Current manuscript has been revised point by point according to the professional comments and constructive suggestions from reviewers. Changes in the manuscript have been identified by page and paragraph, and labelled by red in the manuscript. Following modifications have been done in this revised manuscript.

1. According to the professional comments and constructive suggestions from reviewer 1, we performed new experiments to check the effects of the rest 6 available components on the SH-SY5Y cells treated by sodium nitroprusside, sodium dithionate or potassium chloride. The 6 components were Baicalein (1, 3 and 10 μM), Chrysin (1, 3 and 10 μM), Dauricine (0.3, 1 and 3 μM), Oroxylin A (1, 3 and 10 μM)), Quercetin (1, 3 and 10 μM) and Wogonin (1, 3 and 10 μM), respectively. However, all of them had no neuroprotective effects on SH-SY5Y cells against the damage induced by sodium nitroprusside, sodium dithionate or potassium chloride.

2. The study was done according to ethical standards including ethics committee approval and consent procedure. And the description of “Experiments were in accordance with guidelines for animal care and were approved by Animal Ethics Committee of Chinese Academy of Medical Sciences & Peking Union Medical College” was added to the "Materials and methods" section of the newly manuscript (page5-line142-144).

3. The docking method in the manuscript has been verified. We redocked the ligand in the crystal structure and obtained the RMSD to confirm the feasibility of docking method. Figure 8 has been modified to make them clearer to reveal two inhibitor binding sites in AChE (Fig.8).

4. The structures of all compounds in Table 3 have been drawn in the revised manuscript. The details can be found in Table 3 (page26-32).

Other modifications have also been done to make minor corrections and make our manuscript conform the request of Frontiers in Pharmacology. In addition, we carefully revised the language throughout the article, including number, tense and incomplete sentences. We appreciate for Editors/Reviewers’ warm work earnestly, and hope that the correction will meet with approval.

The comments and critiques of the reviewers have been addressed and itemized as follows:

**Response to the Reviewer 1**

Dear reviewer,

Thank you for your hard work on our manuscript, which we really appreciated. The professional comments and constructive suggestions from you made us realized the defects of our manuscript and inspired us a lot to optimize it. The newly revised manuscript has been revised point by point according to your comments and suggestions. In the following statements, we used “blue” words to introduce what we have revised and modified during the revision following each comment.

Q1: Please summarize the main findings of the study.

Reviewer 1: The authors revealed that Fangchinoline and dauricne might be the potential lead constituents in XXMD for the treatment of AD.

Authors’ response: Thank you very much. Through the China Natural Products Database, TCMSP Database, TCM-Database @Taiwan and literature, a total of 1481 ingredients were finally collected. After ADME/T properties filtering, 908 ingredients were kept for the further study. The prediction results were further validated by *in vitro* experiment, including *in vitro* AChE inhibitory assay and cell-based neuroprotective assay. Among constituents in XXMD, fangchinoline and dauricine might be the potential lead compounds for the treatment of AD.

Q2: Please highlight the limitations and strengths.

Reviewer 1: Strengths: A network pharmacology-based method was applied to predict the potential targets and unconver the material basis of XXMD for the treatment of AD.

Limitations: The availabe compoments of XXMD were only tested for their inhibitory activity towards ACHE, and their activity against other predicted targets related to AD were not disclosed. In additon, the neuprotective activity of the rest 6 avaiable components shoule also be tested.

Authors’ response: Thank you very much for your suggestions. The professional comments and constructive suggestions from you made us realized the defects of our manuscript and inspired us a lot to optimize it. According to the target distribution of potential effective constituents in XXMD, COX-2, ERα and AChE are the major targets of constituents in XXMD linked to the potential treatment of AD. Among them, AChE is the key enzyme in the hydrolysis of the neurotransmitter acetylcholine (Saxena and Dubey 2019). The cholinergic hypothesis suggests that AD arises due to the dysfunction of acetylcholine containing neurons in the brain and most of the clinically used anti-AD drugs preserve acetylcholine inhibiting AChE (Colovic, 2013). AChE is regarded as one of the drug targets for the treatment of AD. Hence, we tested the available 7 compounds interacting with more than 10 AD-related targets in XXMD for their inhibitory activities towards AChE. According to reviewer’s suggestion, we will test their activities against other predicted targets related to AD in the future study.

In addition, your kind reminding helps us realize that it would be very necessary to perform the experiment to identify the neuroprotective activities of the rest 6 available components. The 6 components were Baicalein (1, 3 and 10 μM), Chrysin (1, 3 and 10 μM), Dauricine (0.3, 1 and 3 μM), Oroxylin A (1, 3 and 10 μM)), Quercetin (1, 3 and 10 μM) and Wogonin (1, 3 and 10 μM), respectively. However, as shown in the following figures, all of them had no neuroprotective effects on SH-SY5Y cells against the damage induced by sodium nitroprusside, sodium dithionate or potassium chloride induced toxicity.

Figure 1 The protective effects of Baicalein (A), Chrysin (B), Dauricine (C), Oroxylin A (D), Quercetin (E) and Wogonin (F) against cell injury induced by sodium nitroprusside in SH-SY5Y cells. Results are presented as means ± SEM, n = 3. ### P<0.001 versus each control group.

Figure 2 The protective effects of Baicalein (A), Chrysin (B), Dauricine (C), Oroxylin A (D), Quercetin (E) and Wogonin (F) against cell injury induced by potassium chloride in SH-SY5Y cells. Results are presented as means ± SEM, n = 3. ### P<0.001 versus each control group.

Figure 3 The protective effects of Baicalein (A), Chrysin (B), Dauricine (C), Oroxylin A (D), Quercetin (E) and Wogonin (F) against cell injury induced by sodium dithionate in SH-SY5Y cells. Results are presented as means ± SEM, n = 3. ### P<0.001 versus each control group.

Q3: Please comment on the methods, results and data interpretation. If there are any objective errors, or if the conclusions are not supported, you should detail your concerns.

Reviewer 1: The conclusions are supported by the results and data interpretation in the present work.

Authors’ response: Thank you very much.

Q4: Check List

Is the English language of sufficient quality?

Reviewer 1: Yes.

Is the quality of the figures and tables satisfactory?

Reviewer 1: No.

Does the reference list cover the relevant literature adequately and in an unbiased manner?

Reviewer 1: Yes

Are the statistical methods valid and correctly applied? (e.g. sample size, choice of test)

Reviewer 1: No

Are the methods sufficiently documented to allow replication studies?

Reviewer 1: No

Are the data underlying the study available in either the article, supplement, or deposited in a repository? (Sequence/expression data, protein/molecule characterizations, annotations, and taxonomy data are required to be deposited in public repositories prior to publication)

Reviewer 1: Yes

Does the study adhere to ethical standards including ethics committee approval and consent procedure?

Reviewer 1: Not Applicable

Have standard biosecurity and institutional safety procedures been adhered to?

Reviewer 1: Not Applicable

Authors’ response: Thank you very much for your suggestions. We have carefully revised the English language, figures and tables to improve the quality. In addition, the study was done according ethical standards including ethics committee approval and consent procedure. And the description of “Experiments were in accordance with guidelines for animal care and were approved by Animal Ethics Committee of Chinese Academy of Medical Sciences & Peking Union Medical College” was added to the "Materials and methods" section of the newly manuscript (page5-line142-144).

Q5: Please provide your detailed review report to the editor and authors (including any comments on the Q4 Check List):

Reviewer 1: The Shen's work focuses on the prediction of the potential targets and unconver the material basis of XXMD for the treatment of AD by a network pharmacology-based method. The authors predicted the possible active substane responsible for treatment of AD and the targets of their action. After *in vitro* bioassay, two substances namely fangchinoline and dauricine was found to be AChE inhibtiors, and fangchinoline also showed obvious neuroprotective activity agaisnt sodium nitroprusside-induced toxicity in SH-SY5Y cells. I think this work should be interesting to the readers in the field of AD treatment.

Minor suggestion:

1) The neuprotective activity of the rest 6 avaiable components shoule also be tested.

2) Draw the structures of all compounds in Table 3.

Authors’ response: Thank you very much for your suggestions. The professional comments and constructive suggestions from you made us realized the defects of our manuscript and inspired us a lot to optimize it.

According to reviewer’s suggestion, we performed the experiment to identify the neuroprotective activity activities of the rest 6 avaiable components. The 6 components were Baicalein (1, 3 and 10 μM), Chrysin (1, 3 and 10 μM), Dauricine (0.3, 1 and 3 μM), Oroxylin A (1, 3 and 10 μM), Quercetin (1, 3 and 10 μM) and Wogonin (1, 3 and 10 μM). However, as shown in the following figures, all of them had no neuroprotective effects on SH-SY5Y cells against the damage induced by sodium nitroprusside, sodium dithionate or potassium chloride induced toxicity.

Figure 1 The protective effects of Baicalein (A), Chrysin (B), Dauricine (C), Oroxylin A (D), Quercetin (E) and Wogonin (F) against cell injury induced by sodium nitroprusside in SH-SY5Y cells. Results are presented as means ± SEM, n = 3. ### P<0.001 versus each control group.

Figure 2 The protective effects of Baicalein (A), Chrysin (B), Dauricine (C), Oroxylin A (D), Quercetin (E) and Wogonin (F) against cell injury induced by potassium chloride in SH-SY5Y cells. Results are presented as means ± SEM, n = 3. ### P<0.001 versus each control group.

Figure 3 The protective effects of Baicalein (A), Chrysin (B), Dauricine (C), Oroxylin A (D), Quercetin (E) and Wogonin (F) against cell injury induced by sodium dithionate in SH-SY5Y cells. Results are presented as means ± SEM, n = 3. ### P<0.001 versus each control group.

In addition, the structures of all compounds in Table 3 have been drawn in the newly revised manuscript. The details can be found in Table 3 (page26-32).

Q12: What is the level of revision required based on your comments:

Reviewer 1: Minor revisions.

Authors’ response: Thank you very much.

**Response to the Reviewer 2**

Concerns raised by the reviewer.

Q1: Please summarize the main findings of the study.

Reviewer 2: In this study, the constituents in XXMD formula could interact with 41 targets associated with AD, and cyclooxygenase-2 (COX-2), estrogen receptor α (ER) and acetylcholinesterase (AChE) were the major targets. The constituents in XXMD were found to be linked to the potential AD treatment through multiple AD-related targets. 62 constituents in XXMD were found to interact with more than or equal to ten AD-related targets. Among them, fangchinoline and dauricine might be the potential lead compounds in XXMD for the treatment of AD. Finally, this work demonstrates the feasibility to apply network pharmacology to analyze TCM prescriptions and implicates the therapeutic effects of XXMD on AD.

Authors’ response: Thank you very much.

Q2: Please highlight the limitations and strengths.

Reviewer 2: strengths: (1) the first to elucidate the mechanisms of action for XXMD for AD treatment using the virtual screening and network pharmacology method.

(2) first identified the potential activity of fangchinoline against AD. (3) Presented the feasibility to apply network pharmacology to analyze TCM prescriptions and implicates the therapeutic effects of XXMD on AD.

Authors’ response: Thank you very much for your good evaluation.

Q3: Please comment on the methods, results and data interpretation. If there are any objective errors, or if the conclusions are not supported, you should detail your concerns.

Reviewer 2: The methods, results and data interpretation are enough for this manuscript.

Authors’ response: Thank you very much.

Q4: Check List

Is the English language of sufficient quality?

Reviewer 2: Yes.

Is the quality of the figures and tables satisfactory?

Reviewer 2: Yes.

Does the reference list cover the relevant literature adequately and in an unbiased manner?

Reviewer 2: Yes

Are the statistical methods valid and correctly applied? (e.g. sample size, choice of test)

Reviewer 2: Yes

Are the methods sufficiently documented to allow replication studies?

Reviewer 2: Yes

Are the data underlying the study available in either the article, supplement, or deposited in a repository? (Sequence/expression data, protein/molecule characterizations, annotations, and taxonomy data are required to be deposited in public repositories prior to publication)

Reviewer 2: Yes

Does the study adhere to ethical standards including ethics committee approval and consent procedure?

Reviewer 2: Not Applicable

Have standard biosecurity and institutional safety procedures been adhered to?

Reviewer 2: Not Applicable

Authors’ response: Thank you very much for your suggestions. Experiments in the study were done in accordance with guidelines for animal care and were approved by Animal Ethics Committee of Chinese Academy of Medical Sciences & Peking Union Medical College. We have highlighted it in the newly manuscript (page5-line142-144).

Q5: Please provide your detailed review report to the editor and authors (including any comments on the Q4 Check List):

Reviewer 2: In this manuscript, the authors present evidence that the constituents in XXMD formula could interact with 41 targets associated with AD, and cyclooxygenase-2 (COX-2), estrogen receptor α (ER) and acetylcholinesterase (AChE) were the major targets. The constituents in XXMD were found to be linked to the potential AD treatment through multiple AD-related targets. 62 constituents in XXMD were found to interact with more than or equal to ten AD-related targets. Among them, fangchinoline and dauricine might be the potential lead compounds in XXMD for the treatment of AD. In conclusion, the authors were the first to elucidate the mechanisms of action for XXMD for AD treatment, using the virtual screening and network pharmacology method. This study first identified the potential activity of fangchinoline against AD. This work demonstrates the feasibility to apply network pharmacology to analyze TCM prescriptions and implicates the therapeutic effects of XXMD on AD. In my opinion, this paper could be accepted in Frontiers in Pharmacology after minor revision.

Comments: Please carefully revise the language throughout the article.

Authors’ response: Thank you very much for your good evaluation and kind suggestions. The professional comments and constructive suggestions from you made us realized the defects of our manuscript and inspired us a lot to optimize it. We carefully revised the language throughout the article, including number, tense and incomplete sentences.

Q12: What is the level of revision required based on your comments:

Reviewer 2: Minor revisions.

Authors’ response: Thank you very much for your good evaluation.

**Response to the Reviewer 3**

Concerns raised by the reviewer.

Q1: Please summarize the main findings of the study.

Reviewer 3: This study predicts the potential targets and uncover the material basis of Xiao-Xu-Ming Decoction (XXMD) for the treatment of AD.

Authors’ response: Thank you very much.

Q2: Please highlight the limitations and strengths.

Reviewer 3: Limitations: The detailed mechanism of action of the two acetylcholinesterase (AChE) inhibitors was not revealed. Strengths: Different approaches including calculation methods and in vitro assays were combined to study the effects of Xiao-Xu-Ming Decoction on the treatment of Alzheimer's Disease.

Authors’ response: Thank you very much for your suggestions. The professional comments and constructive suggestions from you made us realized the defects of our manuscript and inspired us a lot to optimize it. As we know, there are no reports available for the anti-AD effect of fangchinoline, we will study the anti-AD action of fangchinoline and the detailed mechanism in the future study.

Q3: Please comment on the methods, results and data interpretation. If there are any objective errors, or if the conclusions are not supported, you should detail your concerns.

Reviewer 3: There are two inhibitor binding sites in AChE, the authors of this study did not reveal the binding site of the two acetylcholinesterase (AChE) inhibitors they found. As there are many docking approaches, such as dock, auto dock, Glide, the authors should redock the ligand in the crystal structure to confirm the feasibility of docking method.

Authors’ response: Thank you very much for your suggestions.

Q4: Check List

Is the English language of sufficient quality?

Reviewer 3: Yes.

Is the quality of the figures and tables satisfactory?

Reviewer 3: Yes.

Does the reference list cover the relevant literature adequately and in an unbiased manner?

Reviewer 3: Yes

Are the statistical methods valid and correctly applied? (e.g. sample size, choice of test)

Reviewer 3: Yes

Are the methods sufficiently documented to allow replication studies?

Reviewer 3: Yes

Are the data underlying the study available in either the article, supplement, or deposited in a repository? (Sequence/expression data, protein/molecule characterizations, annotations, and taxonomy data are required to be deposited in public repositories prior to publication)

Reviewer 3: Yes

Does the study adhere to ethical standards including ethics committee approval and consent procedure?

Reviewer 3: Yes

Have standard biosecurity and institutional safety procedures been adhered to?

Reviewer 3: Yes

Authors’ response: Thank you very much for your good evaluation.

Q5: Please provide your detailed review report to the editor and authors (including any comments on the Q4 Check List):

Reviewer 3: The author should verify the docking method they used is appropriate for the AChE inhibitor binding mode analysis.

Authors’ response: Thank you very much for your suggestion. The professional comments and constructive suggestions from you made us realized the defects of our manuscript and inspired us a lot to optimize it. Reminded by you, in the newly revised manuscript, the CDOCKER module of Dock Ligands in Discovery Studio 2016 was used to do the docking. We redocked the ligand in the crystal structure and obtained the RMSD to confirm the feasibility of docking method. The RMSD of docking method has been shown in the manuscript. The RMSD are shown as follows.

| Name | RMSD (A) |
| --- | --- |
| 4EY7 1 | 0.8926 |
| 4EY7 2 | 0.8664 |
| 4EY7 3 | 0.8869 |
| 4EY7 4 | 0.9133 |
| 4EY7 5 | 0.9014 |
| 4EY7 6 | 0.1939 |
| 4EY7 7 | 1.0025 |
| 4EY7 8 | 1.3355 |
| 4EY7 9 | 2.3338 |
| 4EY7 10 | 2.3560 |

Q12: What is the level of revision required based on your comments:

Reviewer 3: Minor revisions.

Authors’ response: Thank you very much.

Thank you very much for your comments and suggestions.

Yours, sincerely

Li Li, PhD, Associate Professor of Pharmacology

Ailin Liu, PhD, Professor of Pharmacology

Guan-Hua Du, PhD, Professor of Pharmacology

Beijing Key Laboratory of Drug Target and Screening Research

Institute of Materia Medica

Chinese Academy of Medical Sciences and Peking Union Medical College

No.1 Xian Nong Tan Street

Beijing 100050, P.R. China.

Tel/Fax: 86-10-63165184

Email: lili@imm.ac.cn

Email: liuailin@imm.ac.cn

Email: dugh@imm.ac.cn

**Reference**

Colovic, M. B., Krstic, D. Z., Lazarevic-Pasti, T. D., Bondzic, A. M. and Vasic, V. M. (2013). Acetylcholinesterase inhibitors: pharmacology and toxicology. *Curr Neuropharmacol* . 11(3):315-35. doi: 10.2174/1570159X11311030006.

Saxena, M. and Dubey, R. (2019). Target Enzyme in Alzheimer's Disease: Acetylcholinesterase Inhibitors. Curr Top Med Chem. 19(4): 264-275. doi: 10.2174/1568026619666190128125912.
